# When RON MET TAM in Mesothelioma: All Druggable for One, and One Drug for All?

**DOI:** 10.3389/fendo.2019.00089

**Published:** 2019-02-26

**Authors:** Anne-Marie Baird, David Easty, Monika Jarzabek, Liam Shiels, Alex Soltermann, Sonja Klebe, Stéphane Raeppel, Lauren MacDonagh, Chengguang Wu, Kim Griggs, Michaela B. Kirschner, Bryan Stanfill, Daisuke Nonaka, Chandra M. Goparaju, Bruno Murer, Dean A. Fennell, Dearbhaile M. O'Donnell, Martin P. Barr, Luciano Mutti, Glen Reid, Stephen Finn, Sinead Cuffe, Harvey I. Pass, Isabelle Opitz, Annette T. Byrne, Kenneth J. O'Byrne, Steven G. Gray

**Affiliations:** ^1^Thoracic Oncology Research Group, Labmed Directorate, St. James's Hospital, Dublin, Ireland; ^2^Cancer and Ageing Research Program, Queensland University of Technology, Brisbane, QLD, Australia; ^3^Department of Physiology and Medical Physics and Centre for Systems Medicine, Royal College of Surgeons in Ireland, Dublin, Ireland; ^4^Department of Clinical Pathology, University Hospital Zurich, Zurich, Switzerland; ^5^Department of Anatomical Pathology, Flinders University of South Australia, Bedford Park, SA, Australia; ^6^ChemRF Laboratories, Montréal, QC, Canada; ^7^Asbestos Diseases Research Institute, Sydney, NSW, Australia; ^8^Sydney Medical School, University of Sydney, NSW, Australia; ^9^The Commonwealth Scientific and Industrial Research Organization, Brisbane, QLD, Australia; ^10^Department of Histopathology, The Christie NHS Foundation Trust, Manchester, United Kingdom; ^11^Department of Cardiothoracic Surgery, New York University (NYU) Langone Medical Center, New York, NY, United States; ^12^Department of Clinical Pathology, Ospedale dell'Angelo, Venice, Italy; ^13^MRC Toxicology Unit, University of Leicester and Leicester University Hospitals, Leicester, United Kingdom; ^14^HOPE Directorate, St James's Hospital, Dublin, Ireland; ^15^Center for Biotechnology, Sbarro Institute for Cancer Research and Molecular Medicine, College of Science and Technology, Temple University, Philadelphia, PA, United States; ^16^Department of Histopathology and Morbid Anatomy, Trinity College Dublin, Dublin, Ireland; ^17^Department of Thoracic Surgery, University Hospital Zurich, Zurich, Switzerland; ^18^Division of Cancer Services, Princess Alexandra Hospital, Brisbane, QLD, Australia; ^19^Department of Clinical Medicine, Trinity College Dublin, Dublin, Ireland

**Keywords:** Malignant Pleural Mesothelioma, RON, MET, TAM, RTK, MST1, LCRF-0004, BMS-777607

## Abstract

Malignant pleural mesothelioma (MPM) is an aggressive inflammatory cancer with a poor survival rate. Treatment options are limited at best and drug resistance is common. Thus, there is an urgent need to identify novel therapeutic targets in this disease in order to improve patient outcomes and survival times. MST1R (RON) is a trans-membrane receptor tyrosine kinase (RTK), which is part of the c-MET proto-oncogene family. The only ligand recognized to bind MST1R (RON) is Macrophage Stimulating 1 (MST1), also known as Macrophage Stimulating Protein (MSP) or Hepatocyte Growth Factor-Like Protein (HGFL). In this study, we demonstrate that the MST1-MST1R (RON) signaling axis is active in MPM. Targeting this pathway with a small molecule inhibitor, LCRF-0004, resulted in decreased proliferation with a concomitant increase in apoptosis. Cell cycle progression was also affected. Recombinant MST1 treatment was unable to overcome the effect of LCRF-0004 in terms of either proliferation or apoptosis. Subsequently, the effect of an additional small molecular inhibitor, BMS-777607 (which targets MST1R (RON), MET, Tyro3, and Axl) also resulted in a decreased proliferative capacity of MPM cells. In a cohort of MPM patient samples, high positivity for total MST1R by IHC was an independent predictor of favorable prognosis. Additionally, elevated expression levels of MST1 also correlated with better survival. This study also determined the efficacy of LCRF-0004 and BMS-777607 in xenograft MPM models. Both LCRF-0004 and BMS-777607 demonstrated significant anti-tumor efficacy *in vitro*, however BMS-777607 was far superior to LCRF-0004. The *in vivo* and *in vitro* data generated by this study indicates that a multi-TKI, targeting the MST1R/MET/TAM signaling pathways, may provide a more effective therapeutic strategy for the treatment of MPM as opposed to targeting MST1R alone.

## Introduction

Malignant Pleural Mesothelioma (MPM) is an aggressive inflammatory cancer, associated with asbestos exposure (1). The vast majority of patients present at an advanced stage, and as a consequence overall survival is dismal, with most patients dying within 1 year of diagnosis (2, 3). Conservative estimates suggest that 43,000 people die from this disease each year (4), however the actual number is probably much greater (5, 6). Although, there have been some recent advances in this disease, current standard of care (combination of pemetrexed and cisplatin chemotherapy) (7) is non-curative and results in a response rate of ~40% (8). Consequently, there is an urgent clinical need to identify novel therapeutic avenues in this disease to improve patient outcomes.

Receptor tyrosine kinases (RTK) are critical signaling mediators involved in key cellular regulatory pathways such as proliferation and apoptosis (9). Several studies have demonstrated that c-MET, Axl, EGFR, ErbB2, ErbB3, IGF1R, and PDGFRβ RTKs are active in MPM (10–14), and that targeting c-MET has anti-proliferative activity in this disease (10).

Récepteur d'origine nantais (RON/MST1R) is a member of the MET proto-oncogene family, is involved in the development of epithelial, bone, and neuro-endocrine tissues (15), and is essential for complete embryonic development (16). A heterodimeric protein, MST1R (RON) is composed of a transmembrane 150 kDa β chain and a 40 kDa extracellular α chain linked by a disulphide bond (17). The main ligand for MST1R (RON) is Macrophage Stimulating 1 (MST1), a member of the Kringle family (18). Binding of MST1 (HGFL) results in auto-phosphorylation and dimerization of MST1R (RON) (18). A number of MST1R (RON) transcripts have been identified, with the two most common having been identified in normal and cancerous tissues including breast, prostate and lung (18), coding for either full-length MST1R (RON) (flMST1R) or a shorter transcript called short-form (sfMST1R) (19). sfMST1R is constitutively phosphorylated and results in a more aggressive cancer phenotype in breast cancer (19). A number of additional MST1R (RON) variants have been identified, many of which are constitutively active and have oncogenic potential including RONΔ165 (also known as ΔRON), RONΔ160, and RONΔ155 amongst others (18).

Once activated MST1R (RON) can signal through MAPK, NF-κB, Src, PI3K/Akt (20), and β catenin (18). Pathological activation of MST1R (RON) is involved in migration, invasion, and the promotion of metastases (20, 21). The potential influence of MST1R (RON) in oncogenesis is further enhanced by the fact that it has now been shown to form heterodimers with other receptors, such as IGF1R, EGFR, and MET with resulting effects on cellular migration, invasiveness and oncogenic transformation (11, 12, 19, 22). Additionally, MST1R (RON) sustains MET oncogene addiction (23), while EGFR dependence in non-small cell lung cancer (NSCLC) involves MST1R (RON) (24).

The TAM receptors, comprising three RTKs, Tyro3, Axl, and Mertk generally play important roles within the immune, reproductive, hematopoietic, vascular, and nervous systems (25). Altered TAM signaling is thought to contribute to chronic inflammatory and autoimmune disease in humans, and is strongly associated with cancer progression, metastasis, and resistance to targeted therapies (25). Altered expression of AXL has been previously demonstrated in MPM (13, 14). In MPM, signaling through the PI3K/mTOR pathway has been shown to be dependent on coordinated activation of EGFR, MET, and AXL (26).

A number of compounds have been developed to target either the MST1R (RON)/MST1 pathway, or multi-RTK inhibitors designed to bind to the kinase domains of these RTKs and inhibit their activity (some of which can inhibit both MST1R (RON), c-MET, and members of the TAM RTK family) and have recently been reviewed (18, 27). For example, the dual MET/MST1R (RON) kinase inhibitor, LY2801653 which is a type-II ATP competitive, slow-off inhibitor binding to the kinase domains of these RTKs, decreased tumor growth and angiogenesis in the lung cancer setting and demonstrated greater efficacy than crizotinib (28). A five amino acid peptide termed NRWHE is also a dual inhibitor of c-MET/MST1R (RON) (27, 29). RON specific agents have also been developed, such as the monoclonal antibodies Zt/f2 which functions by inducing RON internalization, subsequently reduces RON expression and impairs downstream signaling activation or Narnatumab ((IMC)-RON8) (Eli Lilly) which acts by blocking RON binding to its ligand, macrophage-stimulating protein (MSP), or the small molecule inhibitor of MST1R (RON) called LCRF-0004 (N-(3-fluoro-4-(2-(1-methyl-1H-imidazol-4-yl)thieno[3,2-b]pyridine-7-yloxy)phenyl)-1-phenyl-5-(trifluoromethyl)-1H-pyrazole-4-carboxamide) which selectively binds to kinase catalytic domains of RON but not MET (30–32).

Other small molecule inhibitors have been developed (BMS-777607 and Merestinib), which whilst marketed as MET inhibitors, have strong inhibitory effects on MST1R (RON), Axl, and Tyro3 in an equivalent nM range (33, 34).

As the activation of multiple RTKs is a frequent event in MPM, this study sought to examine and characterize the role of both the MST1/MST1R (RON) and TAM RTK signaling pathways in this disease. In addition, we determined the efficacy of targeting these pathways using either agents that specifically target the MST1/MST1R (RON) pathway ((IMC)-RON8, NRWHE and LCRF-0004), or an agent that targets the MST1/MST1R (RON) pathway, c-MET, Axl, and Tyro3 (BMS-777607).

## Results

### The MST1R (RON)/MET/TAM RTKs Are Frequently Activated in Mesothelioma and Expressed in MPM Cell Lines

**Figure 1:**
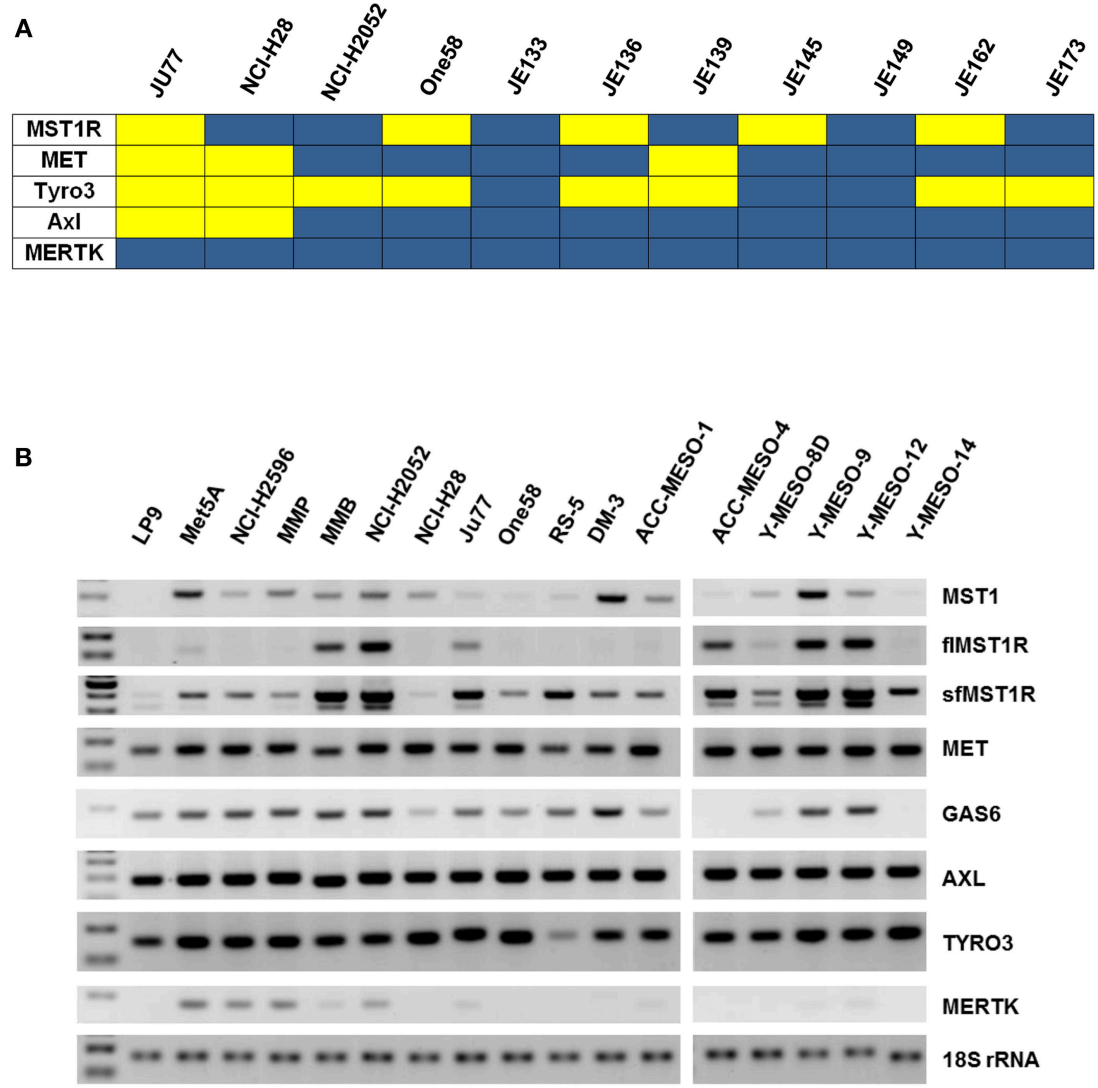
MST1R (RON) is activated in MPM patient samples and cell lines. **(A)** A heat map summarizing the basal phosphorylation levels of the MET (HGFR), MST1R (RON), and the TAM RTKs (TYRO3, AXL, and MERTK) in MPM tumors (*n* = 7) and cell lines (*n* = 4; Ju77, NCI-H28, NCI-H2052, ONE58). Signals with an intensity value greater than the 99% confidence interval of the mean of the 10 negative controls were scored as positive. Yellow indicates high activity and blue indicates low or undetectable kinase activity. **(B)** flMST1R and sfMST1R, MET, MST1, AXL, TYRO3, MERTK, and GAS6 were detected at the mRNA level (standard end point PCR), in a panel of MPM cell lines, which included two normal mesothelial cell lines (LP9 and Met5A) (*n* = 17). 18S rRNA was used as a loading control.

A phospho-RTK array was utilized to screen for activated RTKs in a panel of MPM patient tumor samples (*n* = 7) and cell lines (*n* = 4). Expression data indicated that c-MET (HGFR), MST1R (RON), and members of the TAM receptors (namely Axl and Tyro3, but not MERTK), were often activated in MPM (Figure 1A, Supplementary Figure 1A). We therefore examined the expression of MST1R, C-MET, AXL, and TYRO3 at the mRNA level in a larger panel of MPM cell lines (*n* = 17). Both fl and sfMST1R were robustly detected in the majority of MPM cell lines at the mRNA level (Figure 1B), similar to the expression of C-MET, TYRO3 and AXL (Figure 1B). Additionally, a number MST1R (RON) β chain isoforms were detected at the protein level such as p110 and p80 (Supplementary Figure 1B).

### Overexpression of MST1R/MET/TYRO3 and AXL Is Frequent in Primary MPM

**Figure 2:**
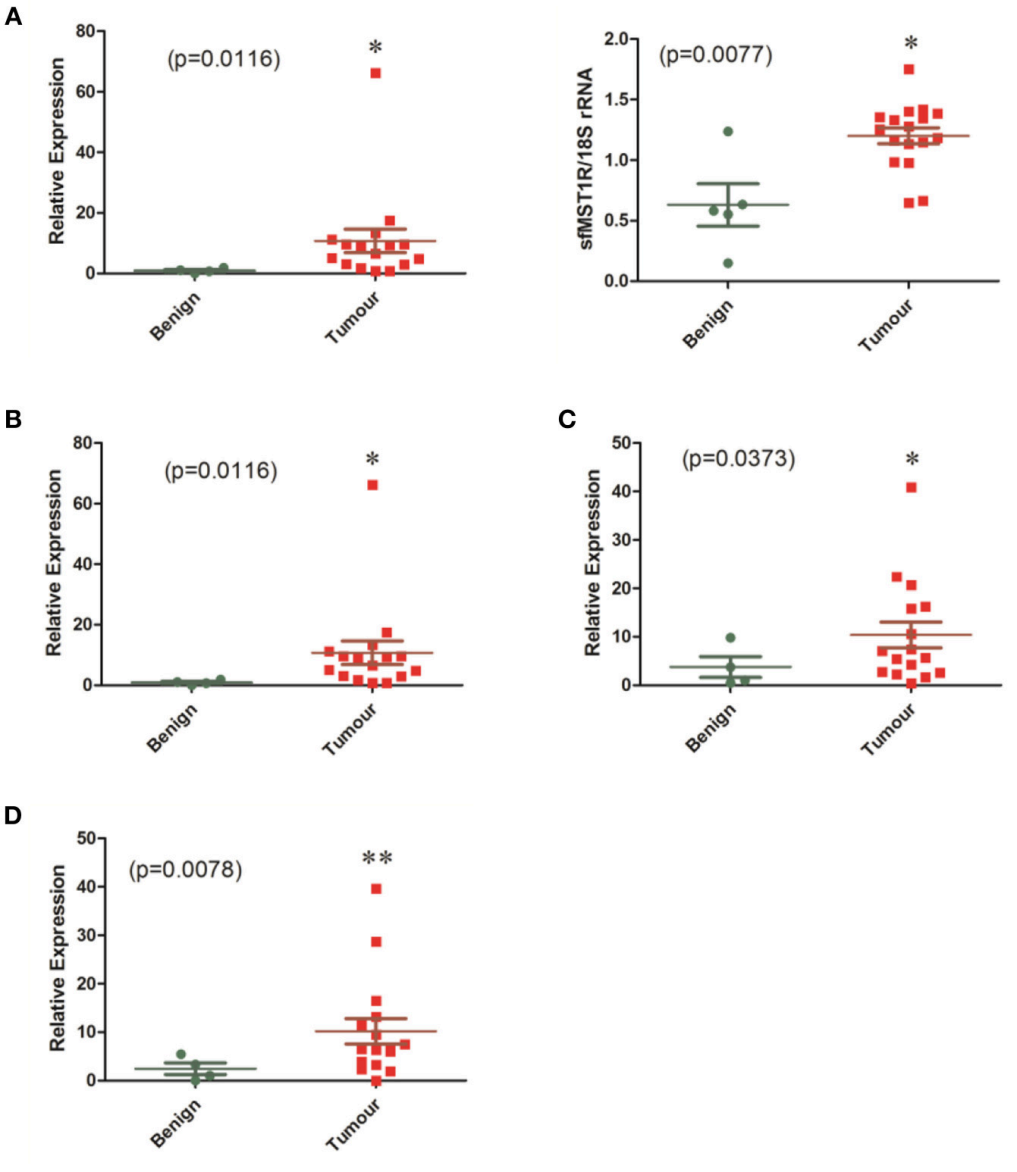
mRNA levels of MST1R/MET/TYRO3 and AXL are elevated in a cohort of MPM patient samples. The mRNA expression of **(A)** MST1R, **(B)** MET, **(C)** AXL, and **(D)** TYRO3 were examined by qPCR or standard end point PCR in a cohort of benign pleura (*n* = 4) vs. MPM patient specimens (*n* = 16). Because detection of sfMST1R utilizes a nested-PCR methodology, densitometric analysis for this gene was used instead on end-point PCR products run on agarose gels, with 18S rRNA serving as a loading control. Significant overexpression of all genes was observed in the MPM specimens compared with benign pleura. Statistical analyses used an unpaired one tailed Student's *t*-test with Welch's correction (*p < 0.05, **p < 0.01).

Strong expression of both sfMST1R and flMST1R mRNA was also observed in fresh-frozen surgically resected mesotheliomas across all histological subtypes (*n* = 17), which was greater than that observed in resected benign tissues (*n* = 5) (Figure 2A, Additional File: Figure S2A). We found the same was true for the other receptors, with significant overexpression of C-MET (Figure 2B, Figure S2B), AXL (Figure 2C, Figure S2C) and TYRO3 (Figure 2D, Figure S2D) in the MPM cohort. When stratified by histology, significant overexpression of sfMST1R and flMST1R, C-MET, TYRO3, and AXL was observed predominantly in the epithelial and biphasic subtypes (Additional File: Supplementary Table S1).

Furthermore, the expression of ΔRON (MST1R) (35), was also detected in the majority of cell lines tested at the mRNA level, and found to be significantly overexpressed in primary patient tumors compared to benign pleura (Figures S3A–C, Supplementary Tables S1, S2). As mutations within the tyrosine kinase domain of MST1R have been identified in Merkel cell carcinoma and Gastroesophageal adenocarcinoma (36, 37), we screened DNA from our panel of MPM cell lines and the 17 fresh-frozen surgical samples for the presence of the R1018G (36) or R1194H (37) mutations. No mutations were found (data not shown). We also examined our panel of MPM cell lines and tumors for the presence of MET exon 14 skipping, and again no mutations were observed (data not shown).

Using Oncomine (38), we queried the expression of the MST1R/C-MET/TYRO3 and AXL receptors in the Gordon et al dataset (39) (Figure S4). We further examined an available mesothelioma TCGA NGS dataset using cBioportal (40) (Figure S4). Finally, we examined the expression of these receptors in a separate series of patients for which microarray data was available (Goparaju and Pass, unpublished) (Figure S4). These *in silico* analyses demonstrate that the MST1R and TYRO3 receptors are significantly overexpressed in mesothelioma (Figure S4), confirming previous data regarding MET (10) and AXL (13, 14), and suggesting that all four receptors may be candidate targets for therapy in MPM.

### MST1 Is Overexpressed in Primary MPM Tumors and Cell Lines

**Figure 3:**
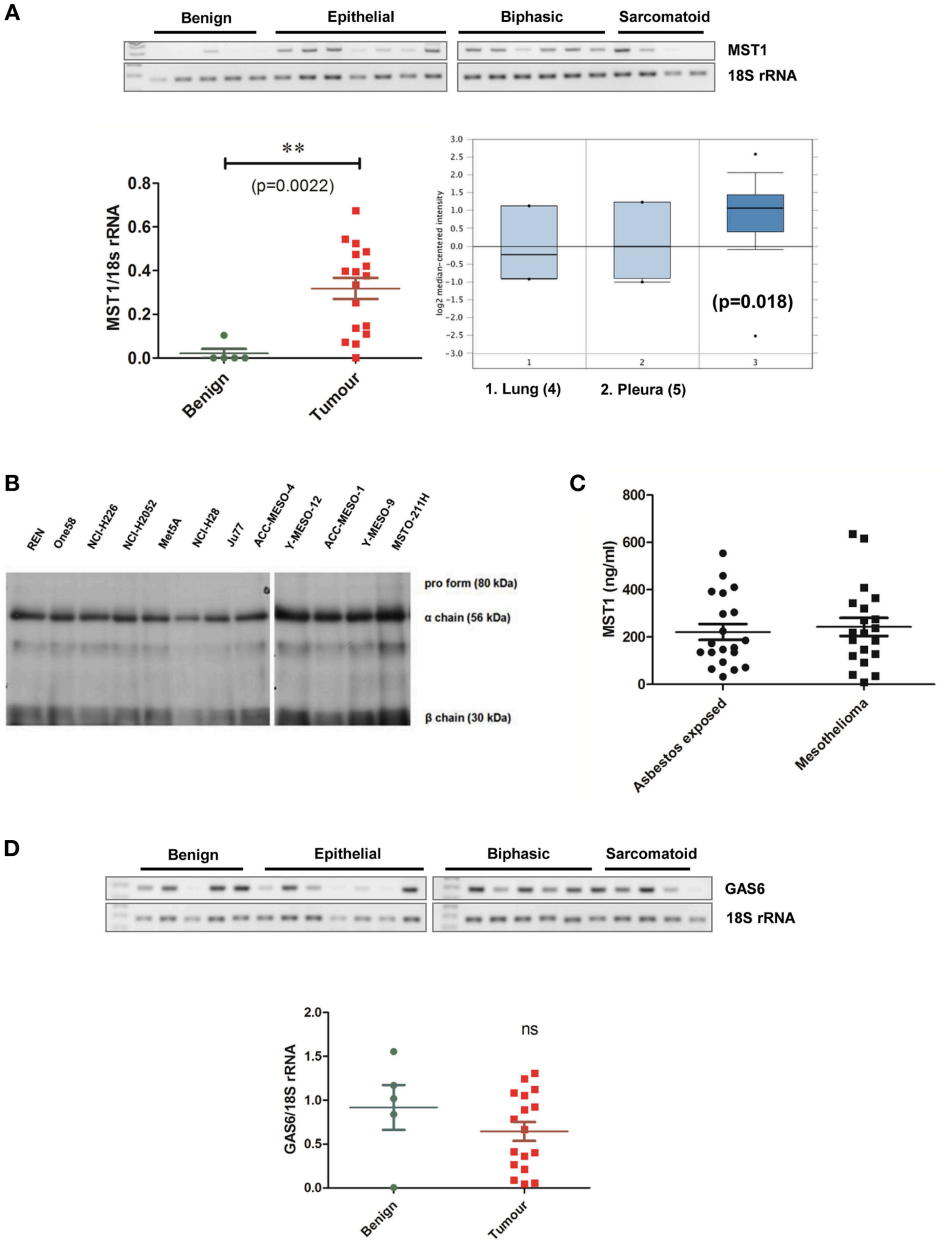
Levels of MST1 are significantly elevated in MPM tissue vs. benign. **(A)** MST1 is overexpressed in primary MPM vs. benign pleura. Analysis of the Leicester samples showed significant (*p* = 0.0022) overexpression in tumors (*n* = 17) vs. benign pleura (*n* = 5) Statistical significance was obtained using a two tailed Student's *t*-test with Welch's correction (**p < 0.01). This data was confirmed using Oncomine analysis of the Gordon MPM dataset (*n* = 49) (37), where MST1 levels were elevated in MPM tumors compared to pleural tissue (*p* = 0.018). For differential gene expression analysis Oncomine uses two sided Student's *t*-test (36). **(B)** MST1 is expressed at protein level in MPM cell lines (*n* = 12). Western blot demonstrating expression of MST1 at the protein level in MPM cell lines. **(C)** Serum MST1 levels were unaltered in patients with primary mesothelioma vs. patients with prior exposure to asbestos (*n* = 20 per group). **(D)** GAS6 mRNA levels were unchanged in the MPM patient cohort compared with benign pleura. β-actin or 18S rRNA is included as a loading control in appropriate images.

We then examined the expression of the ligand for MST1R (RON), MST1 in primary MPM and cell lines. In patient samples, MST1 mRNA was significantly elevated in the tumor specimens (*n* = 17) compared to benign (*n* = 5; *p* < 0.0022; Figure 3A), with significant overexpression observed in the epithelial and biphasic subtypes (Supplementary Table S1). Analysis of the Gordon dataset in Oncomine, subsequently confirmed the significantly elevated MST1 expression in tumor tissues (Figure 3A). MST1 was found to be ubiquitously expressed at the protein level in mesothelioma cell lines (Figure 3B). We also examined the levels of MST1 in patient serums. Elevated MST1 was not observed in patients with mesothelioma compared to at risk (asbestos exposed) patients (Figure 3C). The TAM RTK ligand, GAS6, was also not significantly altered at the mRNA level in patients (Figure 3D).

### MST1R (RON), Tyro3, and MST1 Expression in MPM Tissues

It is well established that c-MET and Axl are overexpressed, activated and have prognostic value in MPM tissues (14, 41), however less is known about other family members. We stained established MPM TMAs (*n* = 132) (42, 43) for expression of MST1R (RON), MST1, and Tyro3.

**Figure 4:**
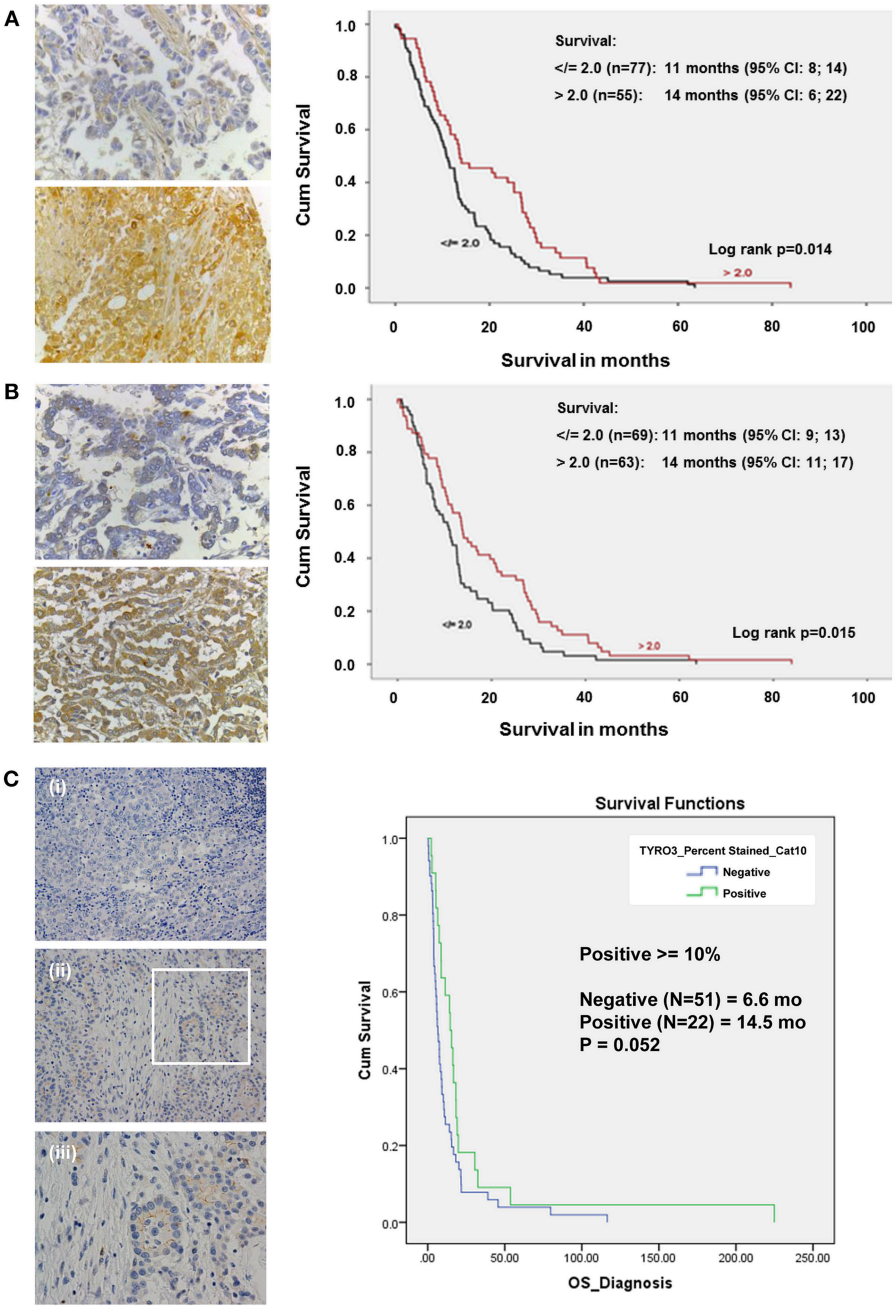
In a cohort of MPM patient samples, elevated levels of MST1R (RON) and MST1 are associated with increased survival. **(A)** Immunohistochemical staining of an MPM TMA for MST1R (Santa Cruz Biotechnology, sc-322, RRID: AB_677390), at (40x) magnification for both focal positive and strongly positive cores. Following scoring by two pathologists, and dichotomized closest to the median into low and high using >2, </=2 global scores, the results were analyzed using Cox regression for survival on (*n* = 132) patients for which clinical data was available. High expression of RON was found to be an independent prognostic factor correlating with an overall increased survival (14 vs. 11 months, *p* = 0.014). **(B)** Immunohistochemical staining of an MPM TMA for MST1 (Santa Cruz Biotechnology, sc-6088, RRID: AB_2235679). At (40x) magnification, both focal positive and strongly positive cores were observed. Following scoring by a pathologist and dichotomized closest to the median into low and high using >2, </=2 global scores, the results were analyzed using Cox regression for survival on (*n* = 132) patients for which clinical data was available. High expression of MST1 was found to be an independent prognostic factor correlating with an overall increased survival (14 vs. 11 months *p* = 0.015). **(C)** Immunohistochemical staining of an MPM TMA for Tyro3 (Abcam ab79778, RRID: AB_10673822) in a patient tumor sample. (i) 20x negative control; (ii) 20x, and (iii) 40x magnification of the inset highlighted in (ii). At (20x and 40x) magnification, both cytoplasmic/membranous staining is observed. When scored and categorized into positive and negative with positive > = 10%, Kaplan-Meier Analyses (with OS from time of diagnosis) was not significant (14.5 vs. 6.6 months *p* = 0.052).

Based on global staining intensity dichotomized closest to the median into low and high, high expression of MST1R (RON) was found to be an independent prognosticator for survival based on Cox regression survival analysis (log rank *p* = 0.014, 14 vs. 11 months) (Figure 4A). Immunohistochemistry for MST1 was subsequently conducted on the same mesothelioma TMA (Figure 4B). Like MST1R (RON), high MST1 expression was found to be an independent prognostic factor for longer survival (log rank *p* = 0.015, 14 vs. 11 months). There was no correlation between gender, histology, or age with global RON or MST1 score. On multivariate analysis, only high expression of MST1 remained significantly associated with increased survival.

Expression of Tyro3 was also examined by IHC in a cohort of MPM patients. In this instance, when staining was categorized into positive and negative (with positive ≥10%), there was a potential trend toward a survival benefit but this was not significant (*p* = 0.052, 14.5 vs. 6.6 months) (Figure 4C).

*In silico* analysis of a mesothelioma TCGA dataset using ProgGene (44) provides further evidence to support these observations (Figure S5). In this data set only MST1R, MST1, or AXL had any survival benefit. In agreement with our data, high expression of MST1R (*P* < 0.022), or MST1 (*P* < 0.05) was associated with better overall survival, which remained significant (*P* < 0.008) under multivariate analysis (Figure S5). High expression of AXL was associated with poorer overall survival (*P* < 0.0000004), in contrast to the data observed by Pinato et al. (14). All other genes examined had no significant survival benefit (data not shown).

As MST1 plays important stimulatory roles with respect to macrophages, TMAs were also stained with the macrophage marker, CD68 (*n* = 130; Figure S6). However, there was no relationship observed between CD68 staining and MST1 or any patient characteristics. Similar results were also observed for *in silico* analysis of the TCGA dataset (Figure S6).

In conclusion, these results suggest that these RTKs are overexpressed in MPM and as such may be suitable candidates for therapeutic intervention.

### Recombinant MST1 Activates a Number of Downstream RTK in MPM

To determine the activity of MST1-MST1R (RON) signaling in MPM, JU77 cells were treated with MST1 (250 ng/mL) for a period of 30 min to 48 h and samples assayed on a phospho-kinase array (46 sites; Figure S7). Densitometry analysis was performed and fold increases were determined relative to their expression at time 0. A significant number of proteins were phosphorylated within 30 min of MST1 treatment with a majority having sustained activation over 48 h. A number of these proteins are critical mediators of the AKT/mTOR and ERK1/2 signaling pathways. In addition, a number of these are downstream mediators, which can be activated by MST1R (RON) through its cross talk with EGFR, IGF-1R and MET (18). We further examined the effect of MST1 on a second MPM cell line (NCI-H226) (Figure S7). When compared across both cell lines a smaller subset of phospho-kinases (predominantly limited to the ERK and AKT signaling pathways) were activated by MST1 (Figure S7). Taken together, these results demonstrate the functionality of MST1R signaling within the MPM setting, affecting a number of critical downstream signaling pathways, and further indicating that MST1R may be a potential target for therapeutic intervention.

### Inhibition of MST1R (RON) Significantly Reduces Cellular Proliferation and Viability

**Figure 5:**
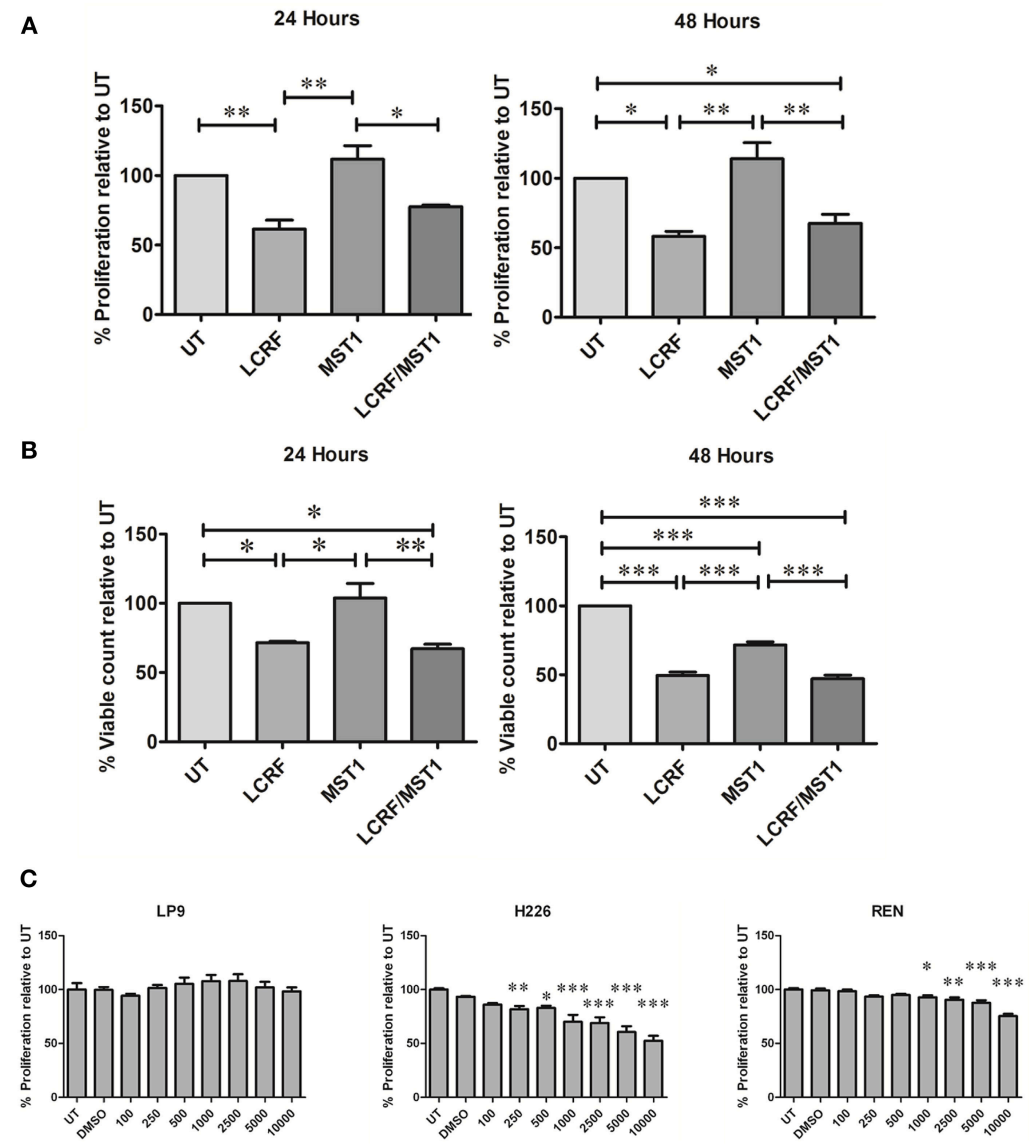
Treatment with LCRF-0004 and BMS-77607 reduces the proliferative capacity of malignant mesothelioma cells. **(A)** Effects of LCRF-0004 (200 nM) and MST1 (250 ng/mL) (alone or in combination) on cellular proliferation as measured using a BrdU proliferation assay. LCRF-0004 significantly affects the proliferative capacity of NCI-H226 cells at 24 or 48 h, while MST1 is unable to rescue the cells from the effect of RON inhibition. **(B)** Cellular viability is also decreased in response to LCRF-0004 treatment (HCS assay) using the NCI-H226 cells. **(C)** Treatment with BMS-77607 resulted in significantly reduced cellular proliferation in both NCI-H226 and REN (MPM cell lines), whilst having no effect on LP9 (normal mesothelial cells). Significance was calculated based on a one-way ANOVA with a post-hoc Tukey's Multiple Comparison test (*p < 0.05; **p < 0.01; ***p < 0.001).

We therefore examined whether MST1R (RON) blockade had any effect on three cell lines (Met5A, JU77 and NCI-H226) using three different approaches (i) LCRF-0004—a MST1R (RON) specific small molecule inhibitor targeting the MST1R (RON) RTK domain, (ii) NRWHE—a five amino acid peptide sequence shown to prevent MST1R (RON) ternary complex formation with CD44 and abrogating RON or MET activation, and (iii) RON8—a humanized monoclonal antibody directed against the extracellular domain of MST1R (and acting to prevent MSP mediated signaling). The dose response effect of these drugs on proliferation is provided in (Figure S8). Intriguingly, LCRF-0004 was the only compound found to significantly reduce the proliferative rate in NCI-H226 MPM cells at both 24 (*p* < 0.01) and 48 h (*p* < 0.05) (Figure S8). Conversely, recombinant MST1 significantly reduced the proliferative capacity of the normal Met5A pleural cell line, while it had no meaningful effect on mesothelioma cancer cells (JU77) (Figure S8). Based on these results, all subsequent experiments utilized LCRF-0004 at 200 nM and MST1 at 250 ng/mL. We subsequently determined the effect of both drugs alone or in combination, on the proliferative capacity of NCI-H226. LCRF-0004 treatment resulted in significant anti-proliferative effects on the cells, which could not be rescued by MST1 (Figure 5A). When cellular viability was examined (Figure 5B), the same observations held at 24 h, however at 48 h, MST1 treatment also resulted in decreased viability (Figure 5B UT vs. MST1). Again, MST1 was unable to rescue the cells from the effect of the LCRF-0004 compound (Figure 5B).

Taken together, these results suggest that specifically targeting the MST1R (RON) RTK domain has anti-proliferative activity.

### Inhibition of MET/MST1R (RON)/Tyro3 and Axl Significantly Reduces Cellular Proliferation

To determine if a multi-targeting approach was also potentially useful, we examined the effect of varying doses of BMS-777607 on cellular proliferation. At 72 h post treatment, a significant decrease in cellular proliferation was observed for MPM cells (NCI-H226 and REN) but not for the normal mesothelial cell line (LP9) (Figure 5C), indicating that targeting all four RTKs is also a potentially useful therapeutic strategy.

### Obstruction of MST1R (RON) Signaling Increases Cellular Apoptosis

**Figure 6:**
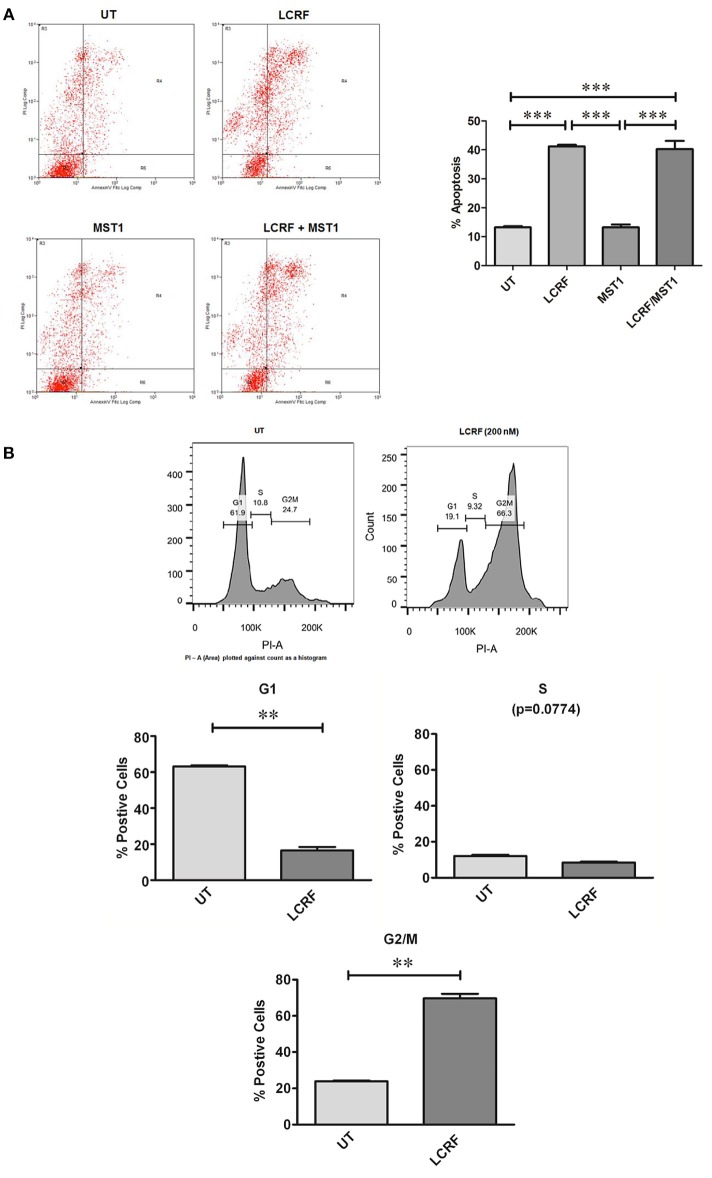
LCRF-0004 promotes apoptosis and alters cell cycle progression in MPM cells **(A)** Inhibition of MST1R (RON) by LCRF-0004 (200 nM) promotes cellular apoptosis in the NCI-H226 cell lines as measured by FACS (48 h post treatment). A representative FACS plots are show with the results graphed underneath (MST1–250 ng/mL). Significance was calculated based on a one-way ANOVA with a *post-hoc* Tukey's Multiple Comparison test. (**p < 0.01; ***p < 0.001). **(B)** FACS analysis determined that LCRF-0004 (200 nM) treatments result in a significant accumulation of cells in the G2/M phase. Significance based on a one-way ANOVA with a Tukey's Multiple Comparisons test (**p < 0.01).

As cellular proliferation and viability were affected by treatments with LCRF-0004, we subsequently examined the effects of this compound with respect to cellular apoptosis. Using a HCS based assay, 200 nM of LCRF-0004 significantly increased cellular apoptosis at both 24 and 48 h for early stage apoptotic cells (apoptotic live) and at 48 h for late stage apoptotic cells (apoptotic dead; Figure S9A). As was the case for proliferation and viability, MST1 was unable to protect cells from the effect of the drug. FACS was also used to assess apoptosis. Representative plots and graphed analysis are shown in Figure S9B. A significant increase in cellular apoptosis was observed at 48 h post treatment (Figure 6A).

### Inhibition of MST1R (RON) Alters Cell Cycle Progression

Given that inhibition of MST1R (RON) can alter cellular proliferation, viability, and apoptosis, this suggested that inhibition of MST1R (RON) might alter cell cycle progression. After 24 and 48 h of drug treatment, significant alterations to cell cycle distribution were observed. Significantly altered cell numbers were determined in either the G0/G1 phase (Figure 6B), or in G2/M (Figure 6B), depending on the assay used, due to the enhanced sensitivity of the FACS. MST1 did not alter cell cycle progression, nor as was the case in other functional assays, could it protect the cells from the effect of LCRF-0004.

### Inhibition of MST1R (RON) *in vivo* Significantly Reduces MPM Tumor Volume

**Figure 7:**
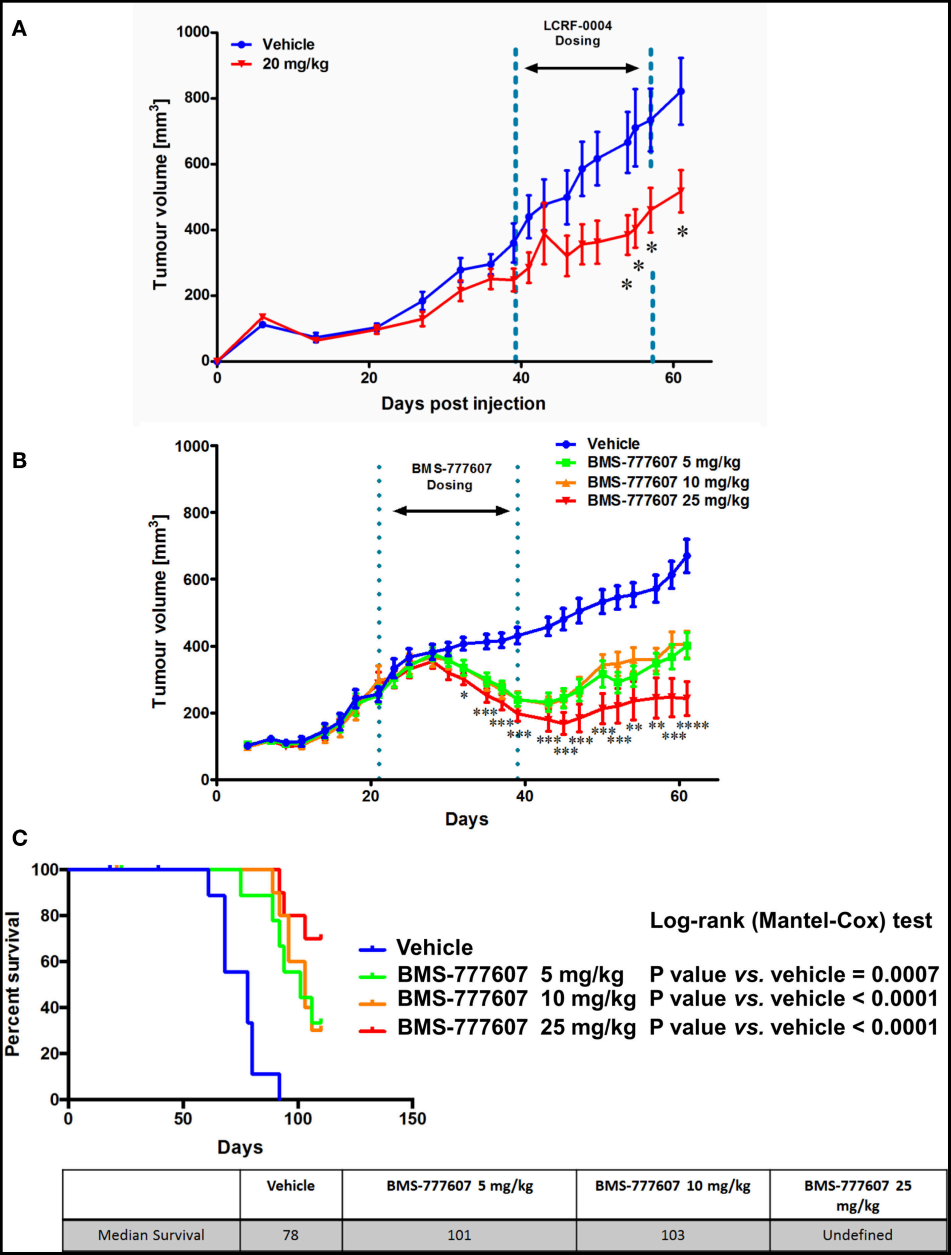
The multi-TKI, BMS-777607, results in superior tumor growth inhibition *in vivo* compared with LCRF-0004. Female nude Balb/c mice were injected sc. with 7 million NCI-H226 cells and randomized in to treatment groups when tumors reached ~200 mm^3^. Tumors were measured using digital calipers and the volume calculated using the modified ellipsoid formula where the greatest longitudinal diameter (b) and the greatest transverse diameter (a) were used (a2 × b × 0.5). DMSO was the vehicle control for both studies. **(A)** Mice were treated with 15 doses of drug (20 mg/Kg; *n* = 10) by i.p. injection over a 3 week period. Treatment started on day 39 and finished on day 57 (Week 1: Days 39–43, Week 2: Days 46–50, Week 3: Days 53–57). Significant inhibition of tumor growth was observed between week 2 and 3. Animal data was analyzed using a two way ANOVA with Bonferroni adjustment with time treated as a discrete variable. Differences were considered significant when *p* < 0.05. **(B)** Mice were treated with 21 doses of drug (5, 10, 25 mg/kg; *n* = 15), by oral gavage, over a 3 week period. Treatment started on day 21 and finished on day 39 (Week 1: Days 21–25, Week 2: Days 28–32, Week 3: Days 35–39) with significant tumor growth inhibition observed during the dosing period, and tumor size measured following removal of drug until the experiment was stopped (day 61). Significance was measured using Restricted maximum likelihood (REML) method (Tukey's Multiple Comparisons test. ns - not significant; *p < 0.05; **p < 0.01; ***p < 0.001; ****p < 0.0001). Further information is available in Supplementary Table S3. **(C)** The animals were monitored up until day 110, and median survival calculated. Median survival was also greater, with it remaining undefined for the 25 mg/kg group. Animal survival analysis was analyzed using Log-Rank (Mantel-Cox) test.

As LCRF-0004 showed promise *in vitro*, we subsequently examined whether LCRF-0004 had similar efficacy in an *in vivo* tumor xenograft model (NCI-H226 cells). Fifteen doses were injected (I.P) over a 3 week period at three different concentrations: 10, 15, and 20 mg/Kg. Tumor measurements were taken every few days and all animals were euthanized when vehicle controls reached 1,500 mm^3^ (Day 61) (Figure 7A). Little difference was observed in the lower dosing groups (10, 15 mg/Kg—data not shown), however significant inhibition of tumor growth was observed on days 54 (384 vs. 666 mm3), 55 (404 vs. 710 mm^3^), 57 (460 vs. 734 mm^3^), and 61 (517 vs. 821 mm^3^) at the highest dose given (20 mg/Kg vs. vehicle). These days correspond to week three of treatment, when mice were receiving their 11th dose of the drug on day 54. To our knowledge, this is the first data to demonstrate the *in vivo* efficacy of MST1R (RON) blockade using LCRF-0004 in a murine model of mesothelioma.

### Combined Inhibition of MST1R/MET/Tyro3 and Axl Is Superior to Inhibition of MST1R Alone

Two orally bioavailable small molecule inhibitors have recently been developed (BMS-777607 and Merestinib), which whilst marketed as MET inhibitors, have strong inhibitory effects on MST1R (RON), Axl, and Tyro3 in an equivalent nM range (33, 34). We therefore examined the effect of BMS-777607 in our NCI-H226 *in vivo* tumor xenograft model. Mice were dosed orally at three concentrations which were administered daily for 3 weeks. All three-drug concentrations showed significant inhibition of tumor growth during the dosing period (days 21–39), and following removal of drug until the experiment was stopped (day 61) (Figure 7B). When survival was assessed, the median survivals were as follows: 5 mg/Kg (101 days) vs. vehicle (78 days) *p* = 0.0007; 10 mg/Kg (103 days) vs. vehicle (78 days) *p* < 0.0001; 25 mg/Kg (undefined) vs. vehicle (78 days) *p* < 0.0001 (Figure 7C).

## Discussion

Receptor tyrosine kinase (RTK) oncogenic signaling networks are becoming increasingly important, particularly in the light of oncogene addiction and/or resistance to drug therapy. Also known as the “Achilles' heel” of cancer, oncogene addiction basically describes a situation where the survival and proliferation of a cancer is dependent upon an overactive oncogene(s) or its downstream pathway(s). Consequently, targeted disruption of this “addicted” oncogene/pathway leads to growth arrest and programmed cell death (45, 46). Using a phospho-RTK array, we screened a number of primary tumors and cell lines for activated receptors. From this screen we identified the c-MET RTK family, and members of the TAM RTK family (Tyro3, Axl, MERTK), as being frequently activated in MPM. While it is well established that the Hepatocyte Growth Factor Receptor (MET) is frequently overexpressed and activated in mesothelioma (10), to our knowledge this is the first indication that it's less well-studied family member, MST1R (RON) is also frequently activated in MPM (Figures 1, 2).

MST1R (RON) can interact with both IGF-1R (47) and EGFR (22) and it is interesting to note that all three were found activated in 2/6 patient samples in our phosho-RTK array analysis, while 3/6 samples demonstrated co-activation of MST1R (RON) and EGFR (Supplementary Figure S1). In this regard, EGFR and MST1R (RON) have been linked to EGFR dependence in NSCLC, as has IGF1R (24, 48, 49).

In this study we have shown that the MST1R, C-MET, TYRO3, and AXL receptors are overexpressed in mesothelioma tumors (Figure 2), as is MST1 (Figure 3), and independently validated these results in various other available datasets (Figures S2–S4).

High expression of MST1R (RON) and its' associated ligand (MST1) is linked to longer survival (14 vs. 11 months) (Figure 4). MST1 is an independent prognostic factor for longer survival and in multivariate analysis only MST1 remained significant for prognosis (Figure 4B). *In silico* analysis of the TCGA mesothelioma dataset confirmed that high expression of MST1R and MST1 was associated with longer survival (Figure S5).

Whilst surprising, higher expression of individual RTKs often correlates with better prognosis. For example, in MPM, overexpression of EGFR and plasma cell membrane MET correlate with better survival (41, 50). Furthermore, high expression of MST1R (RON) has been linked to cisplatin resistance in ovarian cancer (51), a principle element in the standard first-line chemotherapy for mesothelioma. Despite these confounding issues, targeting of these RTKs may still have potential benefit in the treatment of MPM.

In addition to MST1R (RON) itself, its' ligand, MST1 plays critical roles in inducing the spreading, chemotactic migration, and phagocytosis of macrophages (18). Staining of our TMA for macrophage infiltration did not however find any significant differences, and had no prognostic value (Figure S4), while serum levels of MST1 also showed no significant alterations between unaffected vs. tumor patients (Figure 3C). When mesothelioma cells were stimulated with MST1, strong effects on downstream signaling pathways were observed using antibody based arrays, suggesting that this pathway is both intact and functional in mesothelioma (Figure S5). Furthermore, recombinant MST1 had significant anti-proliferative effects on a normal pleural cell line, whilst having no effect on a malignant cell line (Figure S6).

Given that JU77 cells seem to have a constitutive activation of the RON MET TAM pathways due to the expression of the sfMSTR1 variant (Figure 1 and Figure S1), it could be argued that the responses to MST1 observed in this cell line (Figure S7) do not truly reflect signaling functionality for this axis compared to the responses observed for NCI-H226, and that cell lines with different activation states of the pathway could instead have been used to test the molecules to ensure that JU77 and NCI-H226 are not “special cases,” or alternatively, cell lines with similar activation states or characteristics could have been used to confirm the results.

In this regard, it must be considered that c-Met and RON can be activated by HGF and macrophage stimulating protein (MSP), respectively. Moreover, activated signaling also depends on the availability of adaptor proteins and signaling intermediates or the tendency of the adaptor proteins and signaling intermediates to undergo homodimerization or heterodimerization. We therefore examined the expression of both the MET/MST1R and TAM signaling pathways in both cells and find that all of the ligands and receptors are expressed at the mRNA level in both these cells (Figure S10). The results that we have observed are consistent with other studies investigating the activation state of RTKs in mesothelioma such as the study by Sekido et al. (11), which also observed frequent coactivation of multiple RTKs in MPM cells, but which differed between cell lines. MST1R and c-Met are co-expressed in many types of tumors and functionally crosstalk, forming heterodimers, and phosphorylating each other (52, 53). Experimentally, it has been shown that c-Met has stronger kinase activity than MST1R (54), and thus it is possible that heterodimers might be more efficiently activated than MST1R-MST1R homodimers, and implies that c-Met-MST1R heterodimers can promote the activation of diverse signaling cascades through different platforms (53).

As such the observed effects in both NCI-H226 and Ju77 reflects not only ligand binding of MST1 to full-length MST1R homodimers but also reflects MST1 binding and signaling via diverse RTK heterodimers (for example c-MET/MST1R), and the subsequent effects of targeting RTKs either with TKI- specific or multi-TKIs as shown in this manuscript and discussed below must be considered in this light.

Interestingly, another RTK found to be highly activated in our samples (5/7 tumor samples) was macrophage colony-stimulating-factor-1-receptor MCSFR (CSF1R) (data not shown). In this regard, Cioce et al. (55) recently determined that CSF1R plays important roles in chemo-resistance in MPM. This further strengthens the notion that receptors associated with macrophage signaling may have critical roles in mesothelioma pathogenesis, and it will be interesting in the future to determine if MST1R (RON) could heterodimerize with this RTK.

Given that MST1R (RON) is overexpressed and activated in MPM (Figures 1, 2), and that its ligand is also overexpressed and functional in eliciting downstream signaling in MPM cells (Figure 3 and Figures S7,S10), we subsequently assessed the effects of three agents directed against MST1R (RON). The first two compounds, a five amino acid peptide (NRWHE), and Narnatumab ((IMC)-RON8) a monoclonal antibody being developed by Eli Lilly had no significant responses on mesothelioma cell lines, and were subsequently dropped from further study (Supplementary Figure S8). Indeed, Eli Lilly has discontinued development of Narnatumab (56). The poor responses observed by Narnatumab or NRWHE may be because both of these target elements of the MST1R (RON) receptor at the extracellular or cell membrane level, and as MST1R (RON) has several truncated receptor variants (lacking extracellular elements) including the constitutively active oncogenic sfMST1R (18), this may have limited their efficacy.

In contrast, a small molecule inhibitor of the MST1R (RON) tyrosine kinase domain (LCRF-0004) had significant effects on mesothelioma cellular proliferation and health, and cell cycle progression (Figures 5–7), and additions of exogenous MST1 were unable to abrogate these effects. Following on from this data, we examined if a multi-targeted approach would also prove useful. BMS-777607 significantly reduced the proliferative capacity of REN and NCI-H226, whilst having no obvious effect on LP9 cells (Figure 5C).

The efficacy of both LCRF-0004 and BMS-77607 was determined in an *in vivo* xenograft setting (Figure 7). Combined inhibition of MST1R (RON)/MET/Tyro3 and Axl is superior to inhibition of MST1R (RON) alone, with survival remaining undefined for the 25 mg/kg dose of BMS-777607. The drug optimization of LCRF-0004 is still ongoing and several new analogs have been disclosed (57–59).

**Figure 8:**
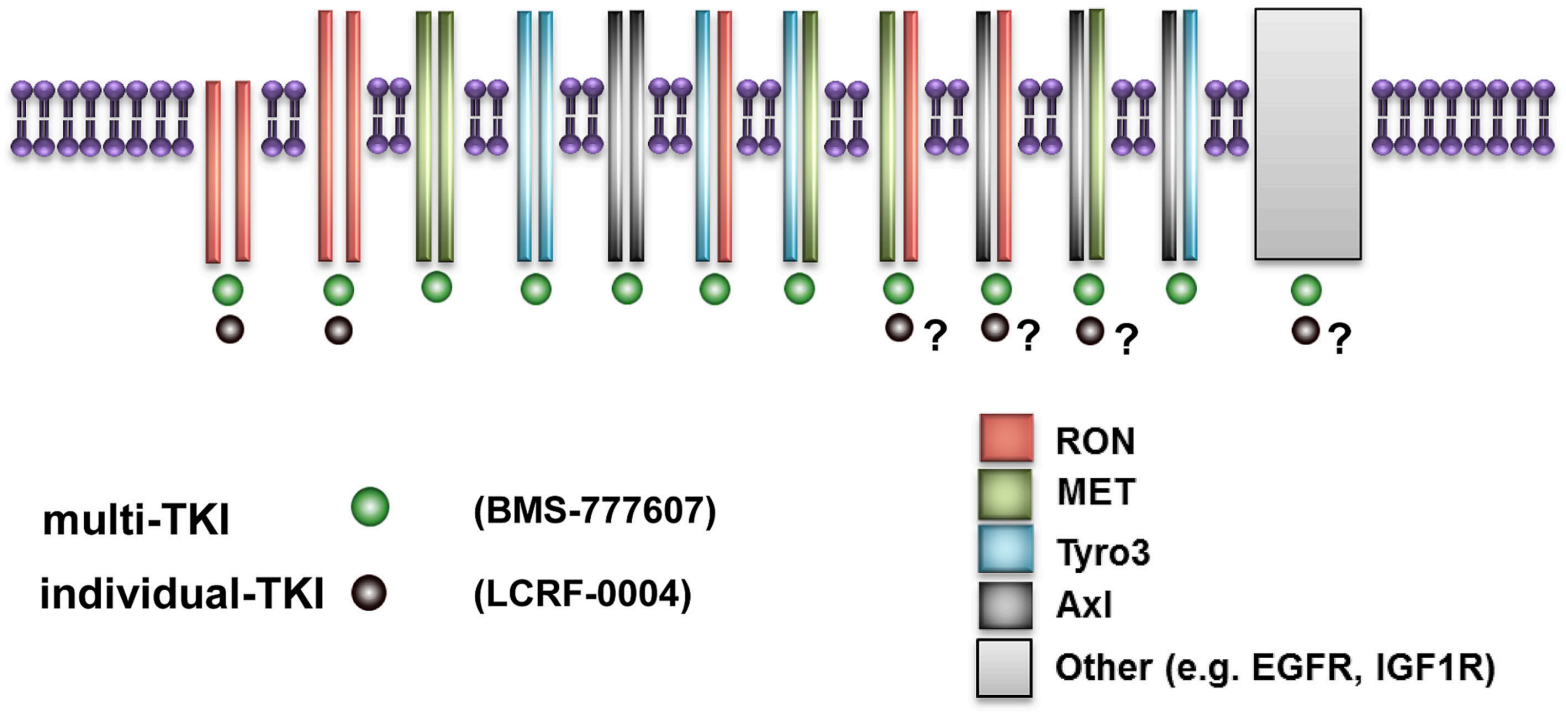
Schematic diagram illustrating putative superior effects of multi-TKI inhibition. This figure represents how small molecule inhibitors such as LCRF which are restricted to a single RTK are limited in their ability to target multi-TKIs if receptor hetero-dimerization occurs. In contrast, small molecule inhibitors such as BMS-777607 can potentially target multiple homo-dimers and heterodimers simultaneously, giving superior results as observed in this study.

Mesothelioma lags behind most solid tumors with respect to having very limited treatment options once standard first line chemotherapy fails. We have identified a novel pathway, the MST1/MST1R (RON) signaling axis, which is overexpressed, activated, and can be targeted pharmacologically in mesothelioma. However, our results suggest that a multi-TKI, targeting the MST1R (RON)/MET/TAM signaling pathways may be a more effective therapeutic strategy for the treatment of MPM as opposed to targeting MST1R (RON) alone. This may in part be due to a potential superiority of multi-TKI to target receptor hetero-dimer formation and cross talk as postulated in Figure 8. One limitation to the current study is that only a single MPM cell line was used for *in vivo* studies. As MPM is a heterogenous disease (60), future studies involving other mesothelioma cell lines or models may further delineate and expand on this possibility.

Another limitation in the current study is that the *in vivo* model used utilized subcutaneous flank xenograft models, which do not truly reflect the growth pattern of malignant pleural mesothelioma. Moving forwards, it may be necessary to use orthotopic animal models to explore whether multi-TKIs are more efficacious than single TKI based targeted therapy.

The results from this study suggest that receptor heterodimerization may be an important element in malignant pleural mesothelioma, particularly with respect to therapeutic targeting. In this regard, whilst c-Met-RON heterodimers occur, their functional relevance of has not been fully delineated. However, some studies have suggested that general loss of MST1R leads to changes in c-Met signaling. For example, it was found that silencing RON in pancreatic cancer cell lines leads to upregulation of c-Met expression and activity (61), whilst another study has demonstrated that oncogenic addition to c-Met requires co-expression of constitutively activated RON that was dependent on transphosphorylation by c-Met (23). This suggests that inhibitors that co-target or simultaneously block the kinase activities of both c-Met and RON might be clinically useful. However, most studies have not considered the possibility that separately inhibiting either c-Met or RON might lead to compensation by the other (53).

The potential for RTK cross talk is not only important in carcinogenesis but may also be critical in drug resistance. A recent study had shown that RTKs such as MET or AXL can work together to drive resistance to EGFR-TKI in NSCLC (62). Additionally, EGFR TKIs can produce off target effects such as the inhibition of phosphorylation on specific residues in both MET and MST1R (RON) (63).

Nevertheless, multi-TKIs are being studied as potential therapeutic targets in cancer. The use multi-TKI such as that used in this study (BMS-777607) which targets various RTKs at equivalent low nanomolar concentrations should be considered a useful tool to test whether receptor heterodimers play critical roles in cancer development and drug resistance. Indeed, while BMS-777607 is marketed as a c-MET inhibitor, one recent study utilized it to therapeutically target AXL in a model of glioblastoma (64).

The issue of receptor heterodimerization as a potential bypass mechanism for lack of response to therapy (or in the development of acquired or intrinsic resistance to therapy) by TKIs is supported by the results obtained in this study. As such future studies may have to include multiple *in vitro* and *in vivo* models of MPM to test these possibilities more rigorously.

## Conclusions

Our results have demonstrated that the MST1R/MET and TAM receptors are overexpressed and may be suitable candidates for therapeutic targeting in MPM. Our results suggest that multi-TKI approaches with small molecule inhibitors targeting multiple TKI domains at low nanomolar concentrations may have greater therapeutic efficacy than agents which are selective for individual RTKs alone. RTK hetero-dimerization is an emerging element in our understanding of the causative mechanisms underpinning resistance to targeted therapies. Multi-TKIs such as the one studied, may have efficacy toward RTK hetero-dimers, and may therefore overcome or limit the development of drug resistance to targeted therapies involving TKIs. Moving forward it may be possible to design a prospective clinical trial in mesothelioma to target multi-RTK signaling pathways targeting the receptors identified in this report.

## Materials and Methods

### Primary Tumor Samples

Surgical specimens were obtained as discarded tumor samples from patients who had undergone extended pleuro-pneumonectomy at Glenfield Hospital, Leicester, UK. Benign specimens were acquired from patients never diagnosed with MPM. Informed consent was obtained from each patient, and the study was conducted after formal approval from the relevant Hospital Ethics Committee (Leicestershire REC references 6742 and 6948). Samples consisted of the following: 5 benign lesions and 17 MPM samples (epithelioid: *n* = 7; sarcomatoid: *n* = 4; biphasic: *n* = 6), details of which are provided in Table 1.

**Table 1:** Patient samples used in this study.

**Sample**	**Pathology (benign, epithelial, biphasic, sarcomatoid)**	**Age**	**Gender**
JE29	Benign—pleural plaque	55	Male
JE30	Benign—pleural plaque	55	Male
JE32	Benign—pneumothorax	30	Male
JE41	Benign—empyema	68	Male
JE48	Benign—pleural plaque	55	Male
JE31	Epithelial	62	Male
JE139	Epithelial	73	Male
JE149	Epithelial	66	Male
JE155	Epithelial	56	Female
JE157	Epithelial	52	Male
JE162	Epithelial	56	Male
JE173	Epithelial	54	Male
JE86	Biphasic	54	Male
JE89	Biphasic	54	Female
JE136	Biphasic	41	Male
JE150	Biphasic	58	Male
JE151	Biphasic	N/A	Male
JE160	Biphasic	60	Female
JE106	Sarcomatoid	74	Male
JE125	Sarcomatoid	64	Male
JE133	Sarcomatoid	59	Male
JE145	Sarcomatoid (desmoplastic)	64	Male

### Ethics Statement

The study was conducted in accordance with the Declaration of Helsinki.

Fresh Frozen Samples: Study was conducted after formal approval from the relevant Hospital Ethics Committee (Leicestershire REC references 6742 and 6948). All subjects gave their written informed consent for inclusion before they participated in the study.

TMAs: Zurich. The construction of the TMA was conducted after formal approval from the relevant Hospital Ethics Committee (Stv.29-2009). Sydney. Ethical approval was obtained from the Human Research Ethics Committee at Concord Repatriation General Hospital, Sydney.

Animal Studies: All animal studies were approved by the relevant institutional Animal Research Ethics Committee (AREC) (AREC789 and AREC1039) at the Royal College of Surgeons in Ireland (RCSI) and were licensed by the Department of Health and Children (B100/3654).

### TMA Immunohistochemistry and Analysis

Two sets of TMAs were used in this study. The first comprises a set of three tissue microarrays (TMA) of *n* = 352 patients with MPM (40), and the second TMA had *n* = 80 MPM patients (41).

Further details on staining, data interpretation, and statistical analysis are provided in Supplementary Materials and Methods S1.

### Cell Culture

All MPM cell lines were maintained in a humidified atmosphere containing 5% CO_2_ in appropriate media supplemented with 10% Fetal Bovine Serum and penicillin streptomycin (500 U/mL). Cell culture reagents were purchased from Lonza (Walkersville, MD, USA). The following MPM cell lines were used in the study: LP9, Met5A, NCI-H2596, MMP, MMB, NCI-H2052, NCI-H28, Ju77, One58, RS-5, DM-3, ACC-MESO-1, ACC-MESO-4, Y-MESO-8D, Y-MESO-9, Y-MESO-12, Y-MESO-14, REN, NCI-H226, and MSTO-211H. ACC-MESO-1, ACC-MESO-4, Y-MESO-9, and Y-MESO-12 were generously provided by Yoshitaka Sekido, (Aichi Cancer Center Research Institute, Japan). NCI-H2052, One-58, and Ju77 cells were provided by Duncan Stewart (University of Leicester, UK). LP-9, MMB, and MMP were a generous gift from Warren Thomas (Royal College of Surgeons in Ireland, Dublin, Ireland). The REN and NCI-H226 cell lines were provided by Dean Fennell (Queen's University, Belfast, Northern Ireland). NCI-H28, and the immortalized non-tumorigenic mesothelial cell line, Met-5A were purchased from the ATCC (LGC Promochem, Teddington, UK). STR profiling of the NCI-H226 was conducted by Source Bioscience (Nottingham, UK) to confirm that the cell line had the correct genotype.

### Drugs and Drug Treatments

Cells were serum starved (0.5% FBS) for 24 h before the addition of drugs and/or recombinant ligand. LCRF-0004 (32) was provided by Dr. Stéphane Raeppel (ChemRF Laboratories, QC, Canada), and dissolved in DMSO. (IMC)-RON8 was provided by Genentech/Eli Lilly (IN, USA) in PBS. NRWHE, a small five amino acid peptide (29) was synthesized for this study by Biomatik (Biomatik Corp., ON, Canada) and re-suspended in sterile water. Recombinant human MST1 was obtained from R & D Systems (MN, USA) and re-suspended in sterile PBS containing 0.1% BSA w/v. BMS-777607 was purchased from Selleck (Munich, Germany) and dissolved in DMSO.

### RNA Isolation and RT-PCR Amplification

Total RNA was extracted using TRI reagent® (Molecular Research Center, OH, USA) according to manufacturer's instructions. Prior to first strand cDNA synthesis, 10 μg of total RNA was pre-treated by digestion with RQ1 DNase (Promega, WI, USA) according to the manufacturer's instructions. cDNA was generated using RevertAid (Thermo Scientific, Leicestershire, UK) and Oligo dT(20) primers (Eurofins MWG Operon, Ebersberg, Germany) according to the manufacturer's instructions. Cell lines and fresh-frozen samples were examined for the expression of full length (fl) MST1R, short form (sf) MST1R, ΔRON, MST1, AXL, TYRO3, GAS6, 18S rRNA, and Beta-actin by either standard end point PCR, using primers and annealing temperatures outlined in Table 2 or qPCR. Cycling conditions for amplification of sfMST1R and flMST1R were as described previously (19). PCR cycling conditions for all others were as follows: 1 min at 95°C, 1 min at the appropriate annealing temperature as outlined in Table 2, 1 min at 72°C, for 35 cycles, with a final extension of 72°C for 10 min. RT-PCR products for each experimental gene and appropriate housekeeping genes (Beta actin or 18S rRNA) were run on 1% agarose gels. Following image capture, product quantification was performed using TINA 2.09c (Raytest, Isotopenmeßgeräte GmbH, Straubenhardt, Germany) densitometry software. The mRNA expression was normalized to loading controls, and was expressed as a ratio of target mRNA expression: loading control expression.

**Table 2:** Primers and associated annealing temperatures.

**Gene**	**Primer sequence**	**Annealing temp. (^**°**^C)**	**Source**
sfMST1R	F: (P1) 5′-CCTCATGACCCTCTTCTGCAGT-3′	56	(19)
	F: (P2) 5′-CAGCAGTGGCACACAGGAT-3′		
	R: (P4) 5′-GCCACCAGTAGCTGAAGACC-3′		
flMST1R	F: 5′-TATCCTGCAGGTGGAGCTG-3′	56	(19)
	R: 5′-ATGAAATGCCATGCCCTTAG-3′		
MST1 (MSP)	F: 5′-TGTTCCAGAACCCACAGCAT-3′	58	This study
	R: 5′-CCCTCAGTGCACATCTCACT-3′		
MST1R Ex13 mutation	F: 5'-CTTCCTCCCAACCTGAATGA-3′	60	(34)
	R: 5′-GGAATCCAGACCATCAATGG−3′		
MST1R Ex17 mutation	F: 5'-TTGCCCACCAACCCACCTGTG-3′	64	(59)
	R: 5′-CACCCCAGCTACTCTGGACTC-3′		
ΔRON	F: 5′- CCTGAATATGTGGTCCGAGACCCCCAG-3′	56	(33)
	R: 5′-CTAGCTGCTTCCTCCGCCACCAGTA-3′		
18S rRNA	F: 5′-GATGGGCGGCGGAAAATAG-3′	58	
	R: 5′-GCGTGGATTCTGCATAATGGT-3′		
Beta actin	F: 5′-TGTTTGAGACCTTCAACACCC-3′	56	(64)
	R: 5′- AGCACTGTGTTGGCGTACAG-3′		
c-METExon14 mutation	F: 5′- TTGCTGGTGTTGTCTCAATATCAAC-3′	58	This study
	R: 5′-GTTAGGATGGGGGACATGTCTG-3′		
c-MET	F: 5′-TACCCCAGCCCAAACCATTT-3′	58	This study
	R: 5′-CAACACCTGTTATTGTGCTCCC-3′		
TYRO3	F: 5′-GAGAGGAACTACGAAGATCGGG-3′	58	This study
	R: 5′-AGTGCTTGAAGGTGAACAGTG-3′		
AXL	F: 5′-GTGGGAGATTGCCACAAGAG-3′	58	This study
	R: 5′-CTTCCCGCAGCTCTGTAAAAC-3′		
MERTK	F: 5′-TTCTCAGTGAGGCAGCGTGC-3′	58	This study
	R: 5′-TGGTCCTGTCTCCAATCGGG-3′		
GAS6	F: 5′-AAGTCGTGGCTCACATCCGC-3′	58	This study
	R: 5′-TCTCCATTAGGGCCAAGGCC-3′		

### Analysis of mRNA Expression by qPCR

Validation of RT-PCR results was subsequently confirmed using SYBR green based quantitative real-time PCR. First-strand cDNA was prepared from 1 μg of total RNA using RevertAid reverse transcriptase (Thermo Scientific) according to the manufacturer's instructions. Prior to reverse transcription, the RNA was treated with amplification grade DNase I (Sigma-Aldrich) according to the manufacturer's instructions. PCR reactions were carried with the primers as shown in Table 2.

MPM patient samples were assessed for suitability for qPCR analysis. Of the original samples (*n* = 4) benign and (*n* = 16), tumor samples were found to be of sufficient quality, and qPCRs were subsequently conducted on these samples using an Illumina Eco qPCR and GoTaq® qPCR Master Mix (Promega) with a 2-step qPCR program having the following cycling parameters:

An initial Polymerase activation of 95°C for 2 min followed by 35 cycles of 95°C 15 s and annealing/amplification 61°C for 1 min. A melting curve analysis was conducted at the end of each PCR using 95°C 15 s, 55°C 15 s, and a final 95°C for 15 s. Data was analyzed using either the default in-built Eco software or imported into EcoStudy (Illumina).

### Mutation Screening

Mutation screening for cMET exon14 skipped cells was conducted by end-point PCR of mRNA from patients and cell lines using primers in Table 1, designed to span exons 13–15. PCR products were run out on a 2% agarose gel.

Mutations in Exon13 and Exon17 of MST1R were analyzed using previously published primers (Table 2) (36, 65). PCR was conducted on patient genomic DNA, and the PCR products were purified and sent for sequencing to a commercial provider (Source Bioscience).

### Protein Isolation and Western Immunoblotting

Total protein was isolated from cell cultures using TRI reagent® (MRCgene) according to manufacturer's instructions. Lysates were separated by SDS/PAGE and subsequently transferred onto a PVDF membrane. Membranes were subsequently probed for expression of the alpha and beta subunits of MST1R (RON α/RON-β), MST1, αβtubulin, or β-actin. Secondary antibodies were HRP labeled and bound antibody complexes were detected using the Supersignal West Pico Chemiluminescent kit (Pierce, IL, USA). Complete details of antibodies and methods are provided in Supplementary file (S1 Supplementary Materials and Methods).

### Immuno-Precipitation of MST1

One mL cell culture supernatant was removed from each cell line, and MST1 immuno-precipitated using anti-MST1 (Santa Cruz) and Protein G PLUS-Agarose beads. Refer to supplementary file S1 (S1-Supplementary Materials and Methods) for further details.

### Protein Arrays

JU77 cell lines were treated with MST1 at a final concentration of 250 ng/mL for a period of 0, 30 min, 24, and 48 h. In an additional experiment, NCI-H226 cells were treated as follows: (i) Untreated, (ii) MST1 (250 ng/mL) for 30 min, (iii) LCRF-0004 (200 nM) for 3 h, and (iv) MST1 and LCRF-0004 combined. Protein lysates were collected and 300 μg assayed on a Proteome Profiler™ Phospho-Kinase Array (R&D Systems) according to the manufacturers' instructions. These arrays contain antibodies to 42–46 kinase phosphorylation sites, spotted on the array in duplicate. In addition, the arrays contain both positive and negative controls. Product quantification was performed using TINA 2.09c (Raytest, Isotopenmeßgeräte GmbH, Straubenhardt, Germany) densitometry software.

### Proliferation Assays

Cell proliferation was measured using either a Cell Proliferation BrdU ELISA (Roche Diagnostics Ltd., Sussex, UK) according to the manufacturer's instructions, or by a Resazurin reduction based assay (66).

For BrDU based assays, cells (JU77, Met5A, and NCI-H226) were seeded at 2.5 × 10^3^/well in a 96-well plate. Following overnight incubation, cells were treated for 24 or 48 h with human recombinant MST1 (0–250 ng/mL), LCRF-0004 (0–200 nM), RON8 (200–1,000 ng/mL), or NRWHE (25–200 ng/mL) or combinations of drug as appropriate. Absorbance was measured on a plate reader at 450 nm with a reference wavelength set to 690 nm. Untreated wells were used for normalization purposes and set to 100%.

For Resazurin based assays cells (LP9, NCI-H226, and REN) were seeded at 3.5 × 10^3^/well in a 96 well plate. Following overnight incubation, cells were serum depleted by replacing the media with fresh media containing 0.5% FBS and left for a further 24 h. Subsequently, cells were treated for 72 h with combinations of drug as appropriate. Resazurin was added after the 72 h treatment period, and following incubation for approximately 4 h at 37°C, fluorescence was measured on a fluorescence plate reader using a 560 nm excitation/590 nm emission filter set.

### Cellular Viability and Apoptosis (High Content Analysis)

NCI-H226 cells were seeded at 2.5 × 10^3^/well in a 96-well plate and adhered overnight. Cells were treated for 24–72 h with human recombinant MST1 (250 ng/mL) and LCRF-0004 (200 nM) alone or in combination. Refer to supplementary file S1 (S1-Supplementary Materials and Methods) for detailed methods on HCA assays.

### Cellular Apoptosis (FACS)

NCI-H226 cells were seeded in 6-well plates at a density of 1 × 10^5^ cells per well and were allowed to adhere overnight. Cells were treated with appropriate concentrations of drug, diluted in cell culture media, for 48 h. Additional details can be found in Supplementary file S1 (S1-Supplementary Materials and Methods).

### Cell Cycle Analysis (Cytell)

NCI-H226 cells were seeded at 2.5 × 10^3^/well in a 96-well plate and adhered overnight. Cells were treated for 24–48 h with human recombinant MST1 (250 ng/mL) and LCRF-0004 (200 nM) alone or in combination. Cells were stained for 45 min at 37°C using the Cytell Cell Cycle Kit (GE Health Bio-sciences), imaged on the Cytell and analyzed, and quantified using the Cell Cycle BioApp (GE Health Bio-sciences).

### Cell Cycle Analysis (FACS)

NCI-H226 cells were seeded at 1 × 10^5^ cells and allowed to adhere overnight. Subsequently, they were treated with either DMSO (vehicle control) or LCRF-0004 (200 nM) for 24 and 48 h. Additional details are provided in Supplementary file S1 (S1-Supplementary Materials and Methods).

### Xenograft Murine Model

All work was performed at the Royal College of Surgeons in Ireland RCSI Biomedical Research Facility (BRF) under Department of Health and Children license #B100/3654 and under RCSI Animal Research Ethics Committee (AREC) approvals (AREC789 and AREC1039). Seven million NCI-H226 cells suspended in 100 ul of serum-free RPMI medium were subcutaneously injected into the right flank of female Balb/c nude mice (Charles River Laboratories, Kent, UK). Tumors were measured using digital calipers and the volume calculated using the modified ellipsoid formula where the greatest longitudinal diameter (b) and the greatest transverse diameter (a) were used (a2 × b × 0.5). When palpable tumor reached approximately 200 mm3, the mice were randomly assigned into appropriate treatment groups. The groups consisted of two separate studies as follows: Study 1—vehicle control (DMSO), LCRF-0004 at 20 mg/Kg (*n* = 10 per group). LCRF-0004 and DMSO were administered via IP injection with each animal receiving 15 doses of treatment, following a Monday-Friday schedule for 3 weeks in total. Study 2—vehicle control (DMSO) (*n* = 14), BMS-777607 at 5, 10, and 25 mg/Kg (*n* = 15 per drug group).

Dosing was determined as follows: LCRF-0004 toxicity data was provided by ChemRF Laboratories which had previously determined that a dose of 20 mg/kg for 14 days resulted in observed anti-tumor activity without any mortality or body weight changes occurring. Using this as a guide, this a 20 mg/kg daily dose was chosen for the experiments shown in Figure 7A.

For the studies using BMS-777607 a similar approach was used. Pre-existing animal data BMS-777607 at doses ranging from 6.25 up to 50 mg/kg has previously been used in GTL-16 human tumor xenografts in athymic mice. The drug was administered orally once daily for 14 consecutive days. The authors reported that the drug was active at all dose levels tested and complete tumor stasis was observed at doses 25 and 50 mg/kg. No overt toxicity was observed at any of these levels as defined by weight loss or morbidity. BMS-777607 at doses 10 and 25 mg/kg has also been used in metastatic mouse tumor model in C3H/HeJ mice. Authors reported that neither 10 nor 25 mg/kg when given orally once daily for 15 days resulted in a significant body weight loss throughout the course of experiment (33, 67). Using these two studies as a guide we used 3 different concentrations of drug to determine which is the most effective in inhibiting tumor growth with the maximum dose at 25 mg/kg.

For both studies drug was administered orally for 3 weeks on a daily basis (21 doses of drug). All animals were sacrificed when vehicle controls and/or tumors reached 15 mm in any diameter.

### *In silico* Analysis

*In silico* analysis was conducted on three additional mesothelioma datasets as follows:

(a) the dataset previously published by Gordon et al. (39), which was interrogated using Oncomine, (b) the TCGA data set, and (c) existing unpublished mesothelioma affymetrix datasets (Goparaju and Pass, unpublished). Data-mining of available mesothelioma datasets were conducted using Oncomine ^1^(38), cBioportal ^2^(40), or PROGgeneV2 (^3^(44)), using their respective default settings.

### Statistical Analysis

All data are expressed as mean ± SEM unless stated otherwise. Statistical analysis was performed with either Prism 5.01 or Prism 6 (GraphPad, Ca, USA) using either paired two-tailed Student's *t*-test or the Mann-Whitney two-tailed *t*-test. One-way analysis of variance (ANOVA) was used where groups in the experiment were three or more. Following ANOVA, post-test analyses utilized either the Tukey multiple comparisons test, or the Dunnett's multiple comparison test. Animal tumor volume data was analyzed using either a two way ANOVA with Bonferroni adjustment with time treated as a discrete variable, or by a restricted maximum likelihood (REML) method using Tukey's Multiple Comparisons test. Differences were considered significant when *p* < 0.05.

## Data Availability

Publicly available datasets were analyzed in this study. This data can be found here: www.oncomine.org.

## Author Contributions

SG and A-MB designed the experiments and wrote the paper. A-MB, DE, MJ, LS, AS, SK, LMacD, CW, KG, and CG performed the experiments. SF, CW, DN, BM, SK, KG, and AS provided pathological and immuno-histochemical support and analysis. MJ and BS conducted the statistical analysis. SR provided access to compound LCRF-0004. LMacD, LM, MJ, AB, GR, MB, DN, DO'D, DF, KO'B, BM, MK, IO, AS, SF, SR, SC, CG, HP, and SG contributed to the sample collection and interpretation of the data. All authors have read and approved the final manuscript for publication.

### Conflict of Interest Statement

SR was employed by company ChemRF Laboratories. The remaining authors declare that the research was conducted in the absence of any commercial or financial relationships that could be construed as a potential conflict of interest.

## Footnotes

^1^Oncomine. Available: www.oncomine.org [Accessed].

^2^cBioportal. Available: www.cbioportal.org [Accessed].

^3^PROGgene. Available: http://watson.compbio.iupui.edu/chirayu/proggene/database/?url=proggene [Accessed].
