# Supplementary material for: When RON MET TAM in Mesothelioma: All Druggable for One, and One Drug for All?
Source: Front Endocrinol (Lausanne). 2019 Feb 26;10:89. doi: 10.3389/fendo.2019.00089 (PMC6399142; doi:10.3389/fendo.2019.00089)
Supplement: Supplementary file 1 [file Presentation_1.pdf]

Supplementary Material

Article Title

When RON MET TAM in mesothelioma: All druggable for one, and one drug for all?

Anne-Marie Baird[1,2], David Easty[1], Monika Jarzabek[3], Liam Shiels[3], Alex Soltermann[4], Sonja Klebe[5], Stéphane Raeppel[6], Lauren MacDonagh[1], Chengguang Wu[4], Kim Griggs[5], Michaela B. Kirschner[7,8], Bryan Stanfill[9], Daisuke Nonaka[10,11], Chandra M. Goparaju[11], Bruno Murer[12], Dean A. Fennell[13], Dearbhaile M. O'Donnell[14], Martin P. Barr[1], Luciano Mutti[15,16], Glen Reid[7,8], Stephen Finn[17], Sinead Cuffe[14], Harvey I. Pass[11], Isabelle Schmitt-Opitz[18], Annette T. Byrne[3], Kenneth J. O'Byrne[1,2,14,19] and Steven G. Gray[1,20*]

1 Thoracic Oncology Research Group, Labmed Directorate, St. James's Hospital, Dublin, Ireland,
2 Cancer and AgeingResearch Program, Queensland University of Technology, Brisbane, QLD, Australia, 3 Department of Physiology and Medical Physics and Centre for Systems Medicine, Royal College of Surgeons in Ireland, Dublin, Ireland, 4 Department of Clinical Pathology, University Hospital Zurich, Zurich, Switzerland, 5 Department of Anatomical Pathology, Flinders University of South Q10 Australia, Bedford Park, SA, Australia, 6 ChemRF Laboratories, Montréal, QC, Canada,
7 Asbestos Diseases Research Institute, Sydney, NSW, Australia, 8 Sydney Medical School, University of Sydney, NSW, Australia, 9 The Commonwealth Scientific and Industrial Research Organization, Brisbane, QLD, Australia, 10 Department of Histopathology, The Christie NHS Foundation Trust, Manchester, United Kingdom, 11 Department of Cardiothoracic Surgery, NYU Langone Medical Center, New York, NY, United States, 12 Department of Clinical Pathology, Ospedale dell'Angelo, Venice, Italy, 13 MRC Toxicology Unit,University of Leicester and Leicester University Hospitals, Leicester, United Kingdom, 14 HOPE Directorate, St James's Hospital, Dublin, Ireland, 15 Department of Medicine, Vercelli Hospital, Vercelli, Italy,
16 Sbarro Institute for Cancer Research and Molecular Medicine, Center for Biotechnology, College of Science 12 and Technology, Temple University, Philadelphia, PA, 19122, USA, 17 Department of Histopathology and Morbid Anatomy, Trinity College Dublin, Dublin, Ireland, 18 Department of Thoracic Surgery, University Hospital Zurich, Zurich, Switzerland, 19 Division of Cancer Services, Princess Alexandra Hospital, Brisbane, QLD, Australia, 20 Department of Clinical Medicine, Trinity College Dublin, Dublin, Ireland

*** Correspondence:**
Steven G. Gray: sgray@stjames.ie; Tel.: +353-1-428-4945

1 Supplementary Materials and Methods

TMA Patients and histological subtypes

Zurich TMA

Tissue specimens of 352 patients with MPM, diagnosed between 1975 and 2004, were sent to the Zurich Pneumoconiosis research group for mineralogical assessment of dust exposure, in particular, asbestos, and later included in this study. The total cohort comprised 114 epithelioid, 192 biphasic and 46 sarcomatoid histotypes. A total of 77% of the specimens were derived from autopsy and 23% from biopsy specimens. Tissue was processed according to the guidelines of the Swiss Society of Pathology. All cases were entirely reviewed for histotype classification by two pathologists (33). Clinical data were retrospectively assessed from medical archives of the different hospitals and the local cancer registries. The construction of the TMA was conducted after formal approval from the relevant Hospital Ethics Committee (Stv.29-2009).

Further clinical data could be retrieved from 206 of these 352 patients (59%), who were mainly males (94%). The median age was 62 years. Asbestos exposure was known in 97 patients (47%). Disease was located in 52% of the patients on the right, 36% on the left and 3% on both sides. As tumour stage was not documentable in most cases, a retrospective staging was performed for 102 patients, based on the surgical pathology reports according to the IMIG staging system, stage T4 being predominant accounting for 71%. Both regional and mediastinal lymph nodes as well as distant metastases were present in 90% of the patients. Only 30% of the patients received therapy, 70% were treatment-naıve. Treatment was surgical for 67 patients and comprised 26 extrapleural pneumonectomies, 16 pleurectomies and 25 palliative procedures, such as talc pleurodesis or tumour debulking. A total of eight patients received only chemotherapy in different combinations, mostly platinum-based. Four patients received radiation therapy. The median overall survival of the 128 patients with complete follow-up data was 11.7 months (95% confidence interval (CI): 10.0—13.6).

Sydney TMA

This series consisted of 80 MPM patients who underwent extrapleural pneumonectomy (EPP) at Royal Prince Alfred (RPAH) or Strathfield Private Hospital (SPH) between 1994 and 2009. All diagnoses of MPM and the subtypes were confirmed by a panel of experienced pathologists from whole sections. A biphasic histological subtype was assigned if both epithelioid and sarcomatoid components were present, and exceeded 10% of the cross sectional area in the slides examined.

TMA construction

Zurich: A set of three tissue microarrays (TMA) with quadruple punches per patient (total n = 1408) was accomplished with a custom-made, semi-automatic tissue arrayer (Beecher Instruments, Sun Prairie, WI, USA), as previously described (34). Sections (4.5-µm thick) of TMA blocks were transferred to an adhesive-coated slide system (Instrumedics, Hackensack, NJ, USA) supporting the cohesion of 0.6 mm array elements on glass.

Sydney: A TMA was generated with five to six cores of 1 mm diameter per patient using an Advanced Tissue Arrayer, ATA-100 (Chemicon, USA). Serial 4-µm thick paraffin sections were transferred to slides for immunohistochemical analysis.

TMA immunohistochemistry

De-paraffined sections were stained either manually or on a Ventana (Ventana Medical Systems, Tucson, AZ, USA), or a Bond automat (Vision Biosystems, Melbourne, Australia), using the following primary antibodies: rabbit poly-clonal anti-MST1R (RON) β antibody (1:100, Santa Cruz Biotechnology, sc-322, RRID:AB_677390); goat poly-clonal anti-MST1 antibody (1:200, Santa Cruz Biotechnology, sc-6088, RRID:AB_2235679); rabbit poly-clonal anti-TYRO-3, (1:300, Abcam ab79778, RRID:AB_10673822), mouse mono-clonal anti-human macrophage, CD68 Antibody (1:50, Dako A/S, clone PG-M1, RRID:AB_2074844). For manual staining citric acid antigen retrieval was utilized.

Detection with appropriate secondary antibodies was performed with Vectastain ABC (Vector Laboratories, Burlingame, CA, USA), DAB (Sigma-Aldrich, St. Louis, MO, USA) and hydrogen peroxide, or with Ultraview Amp (Ventana Medical Systems, Tucson, AZ, USA) or Refine-DAB (Vision Biosystems, Melbourne, Australia).

TMA Data interpretation and statistical analysis

Zurich: The intensity level of immunoreactivity of the MST1R, MST1 proteins and CD68 macrophage was scored semi-quantitatively: 0 (negative), 1 (weak), 2 (moderate) and 3 (strong) in the tumor cell cytoplasm, by two observers (A.S. and C.W.). A mesothelioma-cell-associated signal was considered if three or more cells were positive. A global sum score was created from the four cores and was dichotomised closest to the median into low and high. Clinical data were retrospectively assessed as completely as possible from the medical archives of different hospitals and the local cancer registry. The material of the patients came mainly from autopsies and there was no standardised therapy during this time period. Associations and correlations with clinico-pathologic parameters were examined by chi-squared and Kendall's taub tests, respectively, for both non-dichotomised as well as dichotomised parameters. OS was calculated by the Kaplan-Meier method and survival time differences were compared using the log-rank test. Significant parameters were further analysed by multivariate Cox proportional hazards regression models to test for independency. All analyses were carried out using the SPSS 16.0.1 software package (SPSS Inc., Chicago, IL, USA).

Sydney: The intensity of staining was assessed for Tyro3 was scored by two observers (SK, KG) and stratified for >10% cells stained. OS was calculated by the Kaplan-Meier method and survival time differences were compared using the log-rank test.

Protein isolation and western immunoblotting

Total protein was isolated from cell cultures using TRI reagent® according to manufacturer's instructions. End lysis buffer was 5% SDS (w/v) supplemented with protease inhibitor cocktail (2 mM AEBSF, 1 mM EDTA, 130 µM Bestatin, 14 µM E-64, 1 µM Leupepin, 0.3 µM Aprotinin), sodium orthovanadate (1 mM) and phenylmethylsulfonyl fluoride (87 mg/mL). Primary tissue protein lysates were also extracted using TRI reagent®. Lysates were separated by SDS/PAGE and subsequently transferred onto a pre-activated polyvinylidene fluoride nitrocellulose membrane (PVDF). All antibodies were purchased from Santa Cruz Biotechnology, Inc. (TX, USA) unless noted otherwise. Membranes for MST1R (RON)α, N-20, sc-14627, MST1 N-19, sc-6088), and αβtubulin (CST#2148) were blocked for 1 h in TBST (10 mM Tris–HCl (pH 7.5), 10 mM NaCl, 0.1% (v/v) Tween 20) containing 5% non-fat dry milk powder and incubated with appropriate primary overnight at 4°C in the same buffer (MST1R (RON)α and MST1 (1:200); αβtubulin (1:1000) – 5% BSA). Using the same buffer conditions MST1R (RON) β, C-20, sc-322 and β-actin (Merck Biosciences, Nottingham, UK) were blocked overnight at RT, followed by incubation with primary antibody (MST1R (RON) β (1:200) for 2 h; βactin (1:20000) for 1 h). All secondary antibodies (1:2000 for 1 h with the exception of MST1R (RON) β were the incubation time was 2 h) were HRP labelled and bound antibody complexes were detected using the Supersignal West Pico Chemiluminescent kit (Pierce, IL, USA). As indicated elsewhere αβtubulin or β-actin were used as loading controls.

Immuno-precipitation of MST1

One mL cell culture supernatant was removed from each cell line, 2 µg/mL anti-MST1 (Santa Cruz Biotechnology, sc-6088, RRID:AB_2235679) was added and samples were incubated at 4°C with agitation for 1 h. Subsequently, 20 µL Protein G PLUS-Agarose beads (Santa Cruz) was added and samples were incubated overnight at 4°C with agitation. Samples were centrifuged briefly (2500 rpm at 4°C for 5 min) and supernatant discarded. Samples were washed 4 times in 1 mL RIPA buffer (50 mM Tris HCl: pH 7.4, 150 mM NaCl, 1 mM EDTA, 1% Triton-X 100 (v/v), 0.1% SDS (w/v)) and centrifuged as previously described. After the final wash, beads were re-suspended in 1:1 RIPA: 2X laemmli buffer (0.6 M Tris HCl, 5% SDS (w/v), 10% 2-β-Mercaptoethanol (v/v), 20% glycerol (v/v), 0.01% bromophenol blue (w/v)), boiled for 5 min at 95°C and centrifuged. An aliquot of the resulting supernatant was loaded on to acrylamide gels. HepG2 (hepatocellular carcinoma) supernatant served as a positive control.

Cellular Viability (High content analysis)

NCI-H226 cells were seeded at 2.5 × 103/well in a 96-well plate and adhered overnight. Cells were treated for 24 – 72 h with human recombinant MST1 (250 ng/mL) and LCRF-0004 (200 nM) alone or in combination. Live cells were stained with propidium iodide (PI) (1 µg/mL) and Hoechst (Bisbenzimide H 33342) (5 µg/mL) for 30 min at 37°C. Cells were imaged on a Cytell Cell Imaging System (GE Healthcare Bio-Sciences, PA, USA) and analysed using IN Cell Investigator software (GE Healthcare). Algorithms were set to identify those as PI positive as dead. Various parameters were quantified using these algorithms including cell number and viable cell number.

Cellular Apoptosis (High content analysis)

NCI-H226 cells were seeded at 2.5 × 103/well in a 96-well plate and adhered overnight. Cells were treated for 24 – 72 h with human recombinant MST1 (250 ng/mL) and LCRF-0004 (200 nM) alone or in combination. Live cells were stained with PI (1 μg/mL), Hoechst (5 μg/mL) and AnnexinV (1:40) (Enzo Life Sciences, Inc., NY, USA) for 30 min at 37°C, imaged using the Cytell and analysed using the IN Cell Investigator software. Algorithms were set to identify (i) apoptotic but live as those stained with AnnexinV only, (ii) apoptotic (dead) stained with both AnnexinV and PI, and (iii) necrotic stained with PI only.

Cellular Apoptosis (FACS)

NCI-H226 cells were seeded in 6-well plates at a density of $1x10^5$ cells per well and were allowed to adhere overnight. Following overnight incubation, cells were treated with appropriate concentrations of drug, diluted in cell culture media, for a further 48 h. Where appropriate, control cells were treated with either vehicle or left untreated with media only. Following treatment, culture media was removed, transferred to labelled FACS tubes and placed on ice. Adhered cells were then trypsinised and transferred to corresponding FACS tubes. Cells were pelleted by centrifugation at 1300 rpm for 3 min and the supernatant removed. The cells were washed in 1 mL 1X binding buffer (BB) diluted in ice cold PBS, pelleted by centrifugation and re-suspended in 100 µL BB. Two µL Annexin V (IQproducts) was added to each tube, with the exception of the negative control and media only samples, and cells were incubated at 4°C for 20 min, protected from light. Cells were again washed in 1 mL 1X binding buffer and supernatant removed. Immediately before analysis by FACS, cells were re-suspended in 400 µL BB containing 0.5 µg/mL PI (Invitrogen), except the negative control and FMO for PI for which BB alone was used, and apoptotic cells were analyzed by Flow cytometry, using a CyAn™ ADP flow cytometer and Summit software (Dako, CO, USA).

Cell cycle analysis (FACS)

Cells were seeded at $1x10^5$ in full serum media and allowed to adhere overnight. Subsequently, cells were serum starved (0.5% FBS media) for 24 h and treated with either DMSO (vehicle control) or LCRF-0004 (200 nM) for 24 and 48 h. Cellular supernatants were harvested, as was the PBS wash. Cells were trypsinised and diluted in a further 5 mL PBS and added to appropriate PBS/supernatant tube. Samples were centrifuged for 5 min and pellets re-suspended in 1 mL ice cold 70% EtOH (while slowly vortexing). Samples were stored at -20°C for 20 min and centrifuged. The EtOH supernatant was removed and the pellet washed in 5 mL PBS. Cells were pelleted and re-suspended in a PBS staining solution containing 10 mg/mL RNAse A and 1 mg/mL PI, and incubated at 37°C for 1 h. Samples were run on a BD LSR II flow cytometer (BS Biosciences, San Jose, CA, USA) and analysed using FlowJo vX software (Ashland, OT, USA). Plots were graphed based on PI Area (PI A) versus count as a histogram.

2 Supplementary Figures and Tables

Supplementary Table S1. Stratification of gene expression according to histological subtype (compared to benign), using one-way ANOVA (Dunnett's Multiple Comparison test)

Gene	Epithelioid	Biphasic	Sarcomatoid
flMST1R	NS	NS	NS
sfMST1R	**	*	NS
ΔMST1R	NS	NS	NS
MET	*	*	NS
MST1	*	*	NS
TYRO3	*	*	NS
AXL	*	***	*
MERTK	NS	NS	NS
GAS6	NS	NS	NS

(* p<0.05; ** p<0.01; *** p<0.001)

Supplementary Table S2. Stratification of gene expression according to histological subtype, using one-way ANOVA (Tukey's Multiple Comparison test)

flMST1R	sfRON	ΔMST1R	cMET	MST1		TYRO3	AXL	MERTK	GAS6	
NS	**	NS	NS	NS		NS	*	NS	NS	Benign vs Epithelioid
NS	NS	NS	NS	*		*	***	NS	NS	Benign vs Biphasic
NS	NS	NS	NS	NS		NS	*	NS	NS	Benign vs Sarcomatoid
NS	NS	NS	NS	NS		NS	NS	NS	NS	Epithelioid vs Biphasic
NS	NS	NS	NS	NS		NS	NS	NS	NS	Epithelioid vs Sarcomatoid
NS	NS	NS	NS	NS		NS	NS	NS	NS	Biphasic vs Sarcomatoid

Supplementary Table S3.

Analysis of the effect of BMS-777607 using a Restricted maximum likelihood (REML) analysis (Tukey's Multiple Comparisons test) on *in vivo* tumor volume data.

Day	Treatment (mg/kg)					
	5 mg/kg		10 mg/kg		25 mg/kg	
	Significant?	Adjusted P Value	Significant?	Adjusted P Value	Significant?	Adjusted P Value
4	ns	0.965	ns	0.9631	ns	0.9961
7	ns	0.9452	ns	0.9538	ns	0.9234
9	ns	0.9947	ns	0.9939	ns	0.742
11	ns	0.9882	ns	0.9614	ns	0.8854
14	ns	0.9991	ns	0.9783	ns	0.9929
16	ns	0.9966	ns	0.9766	ns	0.9999
18	ns	0.9788	ns	0.8918	ns	0.9627
21	ns	>0.9999	ns	0.8006	ns	0.8423
23	ns	0.8954	ns	0.8862	ns	0.8259
25	ns	0.8925	ns	0.9406	ns	0.7253
28	ns	0.9969	ns	0.9729	ns	0.8223
30	ns	0.6036	ns	0.7385	ns	0.074
32	ns	0.107	ns	0.1047	**	**0.0022**
35	**	**0.0039**	*	**0.0104**	***	**0.0001**
37	***	**0.0004**	***	**0.0005**	****	**<0.0001**
39	****	**<0.0001**	****	**<0.0001**	****	**<0.0001**
43	***	**0.0001**	****	**<0.0001**	****	**<0.0001**
45	***	**0.0003**	***	**0.0002**	****	**<0.0001**
47	**	**0.001**	***	**0.0009**	***	**0.0001**
50	**	**0.0042**	**	**0.0044**	***	**0.0002**
52	***	**0.0003**	**	**0.0045**	***	**0.0004**
54	***	**0.0005**	**	**0.0066**	**	**0.0015**
57	**	**0.0027**	**	**0.0051**	**	**0.0021**
59	**	**0.0018**	**	**0.0069**	***	**0.0005**
61	**	**0.0035**	**	**0.0039**	****	**<0.0001**

(ns – not significant; * p<0.05; ** p<0.01; ***p<0.001; ****p<0.0001)

3. Supplementary Figures

Figure S1. Identification of MST1R/MET/TYRO3 and AXL as activated RTKs and expression of MST1R in MPM cell lines

(A) Phospho-RTK screen was performed using Phospho-Kinase Proteome Profiler arrays (R & D Systems). Treatment values were compared with the 0 min time point to determine whether a protein was up or down regulated. An arbitrary cut-off of ± 20% change was set for increased or decreased activity, and the results are shown in tabulated heat map form. Green indicates decreased kinase activity, while red indicates increased. (MPM cell lines: n=4 (JU77, NCI-H28, NCI-H2052, One58); MPM patient cohort: n=7).

(B) Western blot images of the MST1R (RON) α and MST1R (RON) β chains. The MST1R (RON) β antibody used, because of its epitope, detected all the MST1R β chain variants, including such as p110 and p80. αβ tubulin was used as a loading control.

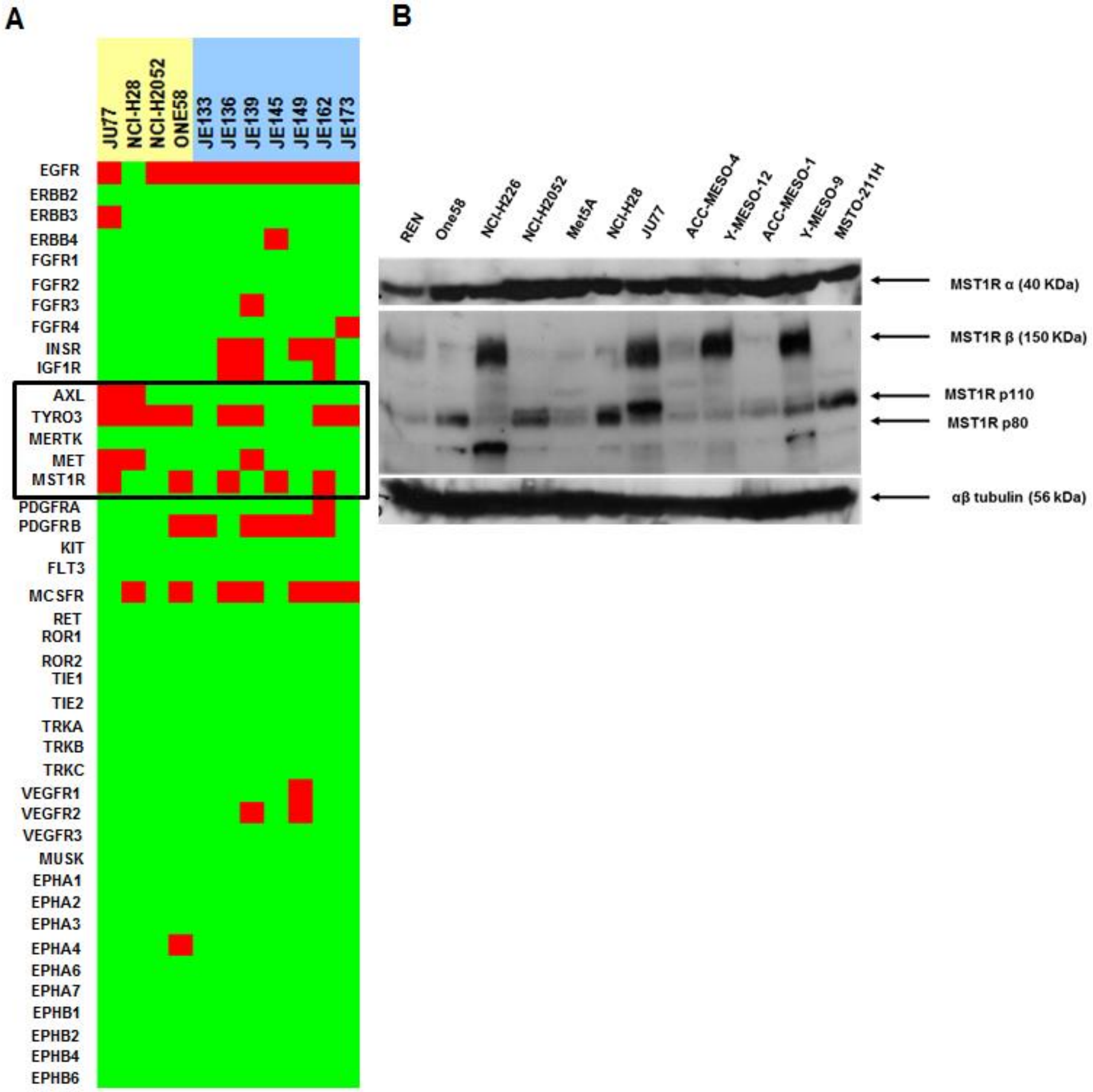

Supplementary Figure 1

Figure S2. Expression of MST1R/MET/TYRO3 and AXL in MPM tissues.
mRNA levels of (A) flMST1R, sfMST1R (B) MET, (C) AXL and (D) TYRO3 are elevated in a cohort of MPM patient samples. Expression levels were quantified using end-point PCR with 18S rRNA serving as the control gene, and analysed semi-quantitatively by densitometric analysis. Significant overexpression of all genes was observed in the MPM specimens (n=17) compared with benign pleura (n=5). Statistical analyses used an unpaired one tailed Student's t test with Welch's correction (*p<0.05, **p<0.01).

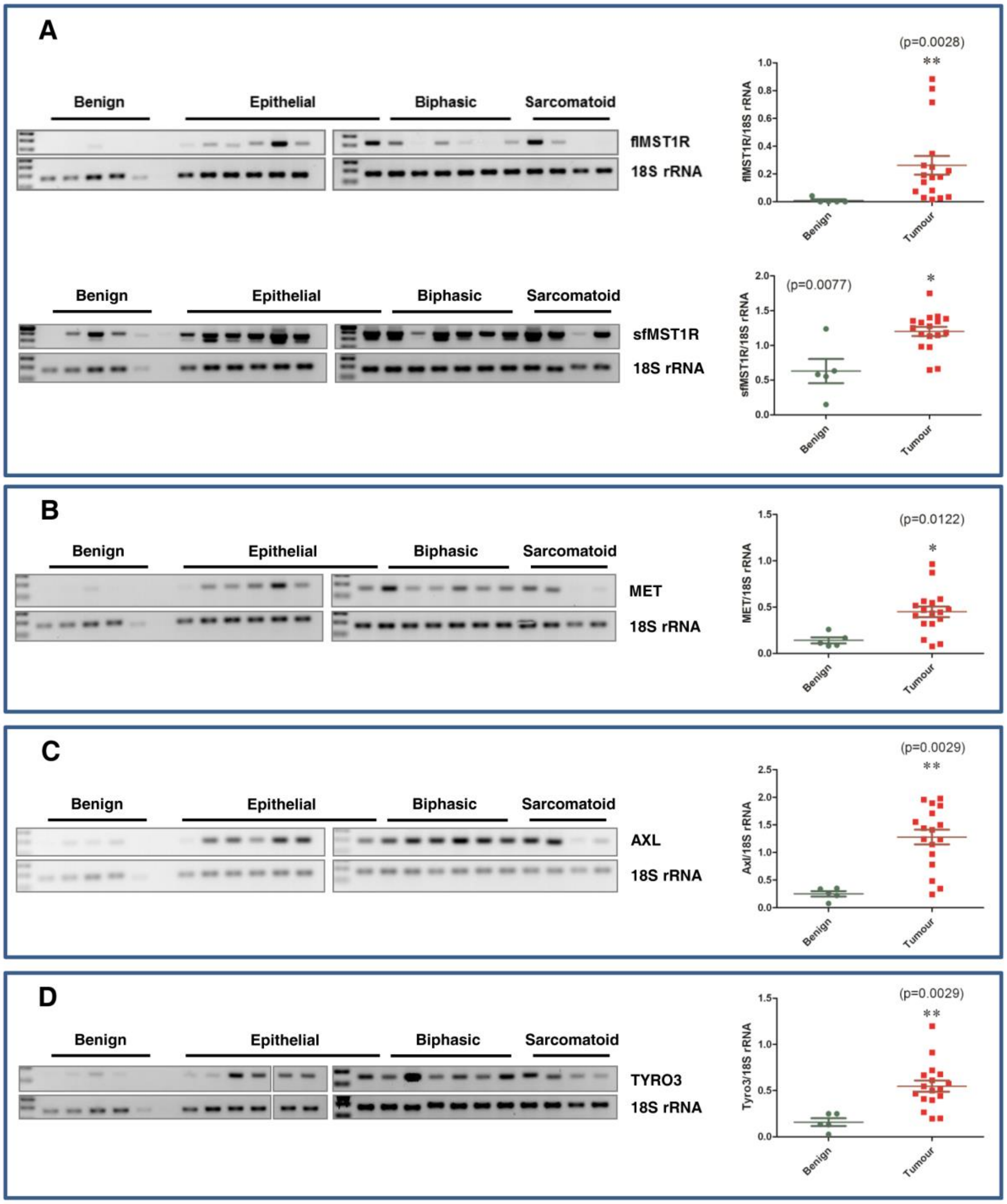

Supplementary Figure 2

Figure S3. Expression of ΔRON in MPM cell lines and tissues.
Expression of ΔRON was assessed using standard end point PCR in a panel of MPM cell line and a cohort of patient tissues.
(A) ΔRON is expressed in various MPM cell lines.
(B) ΔRON is expressed in primary MPM tissue (Benign: n=6; MPM: n=17).
(C) ΔRON is significantly ($p<0.05$) overexpressed in tumors versus benign pleura. Statistical significance was measured using a Mann-Whitney two-tailed t-test (*$p<0.05$).

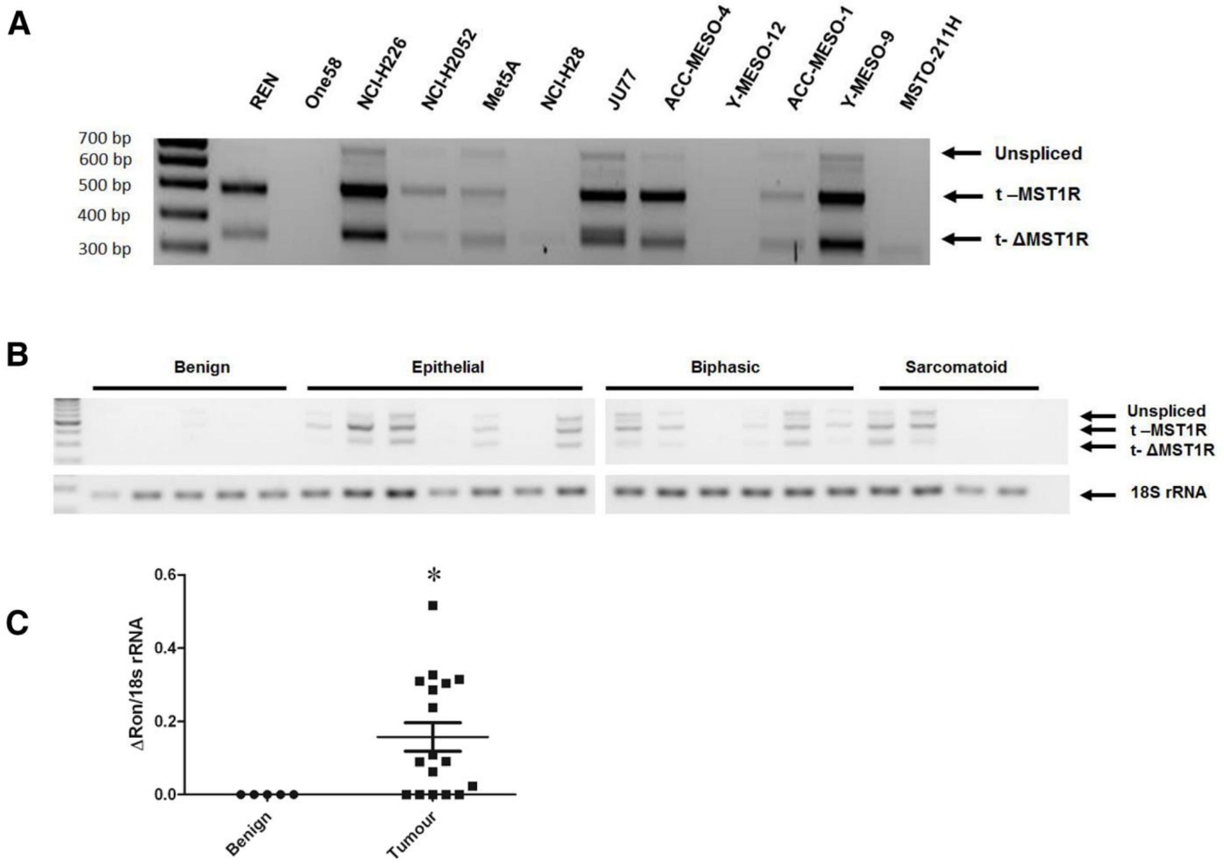

Supplementary Figure 3

Figure S4. Validation of MST1R/MET/TYRO3 and AXL expression in MPM cells

(A-D) Oncomine analysis for MST1R, MET, TYRO3 and AXL (n=49). All genes bar AXL were significantly increased in MPM tissue compared with benign pleura.

(E) Analysis of the TCGA NGS dataset comprising 87 samples using cBioportal. Several samples show elevated mRNA for the various receptors.

(F) Analysis of additional microarray data sets (Goparju and Pass, unpublished) showing significantly increased levels of the receptors in MPM (sample numbers vary by gene) for MST1R, MET, TYRO3 and AXL.

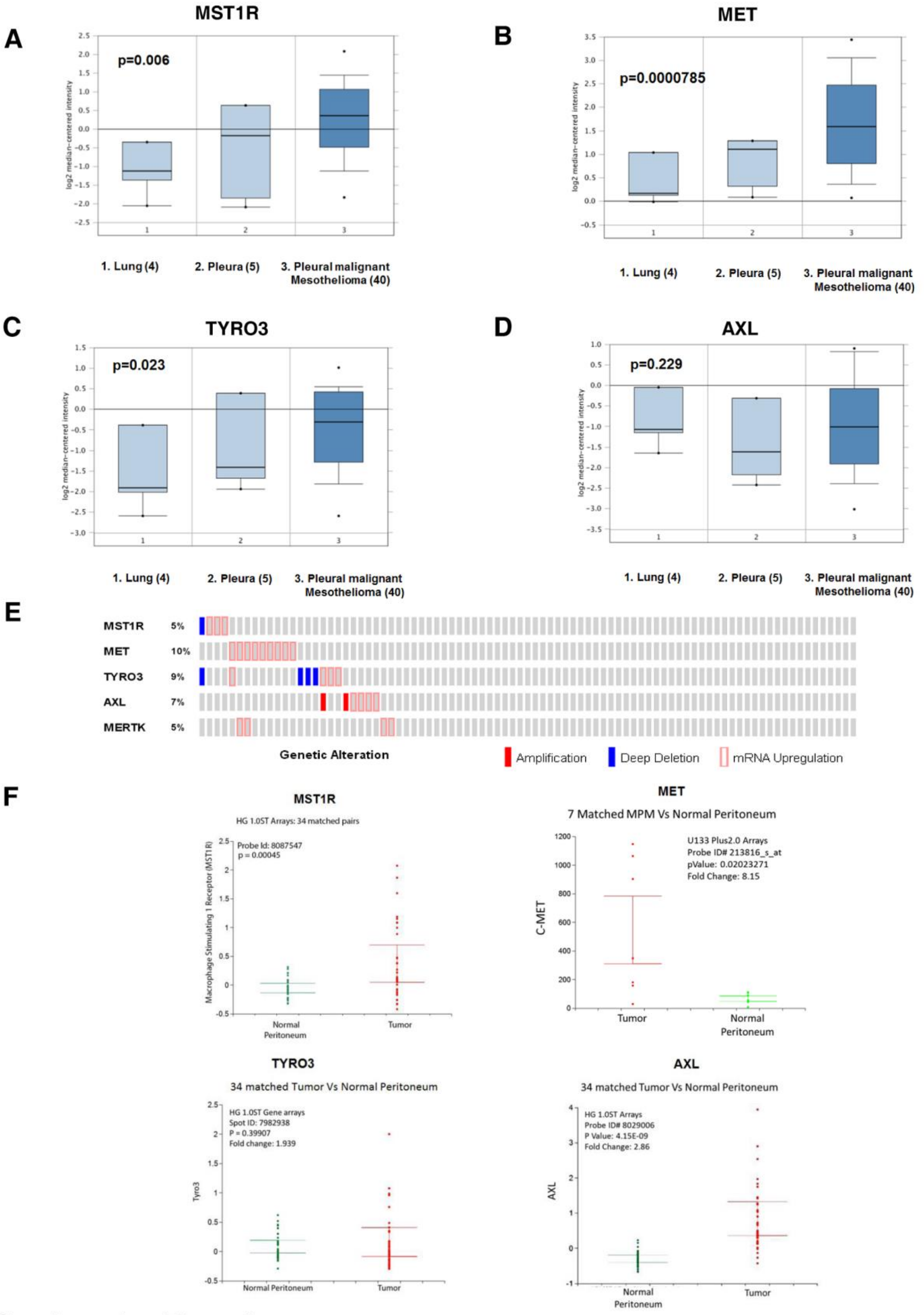

Supplementary Figure 4

Figure S5. Overall Survival analysis in a TCGA mesothelioma dataset using PROGgene. Overall survival for (A) MST1R (univariate); (B) MST1 (univariate); (C) MST1R and MST1 (multivariate); and (D) AXL (univariate) using the PROGgene websuite, showing that expression levels of each have significance for OS.

A

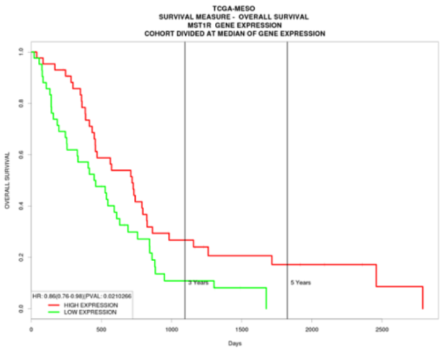

MST1R

HAZARD RATIO	LCI (95%)	UCI (95%)	P VALUE
0.86	0.76	0.98	0.0210266130874606

CATEGORY	SAMPLES	NO OF EVENTS	MEDIAN SURVIVAL	LOW CONF INT (95%)	UPP CONF INT (95%)
HIGH	43	35	719	457	826
LOW	42	38	454	255	689

B

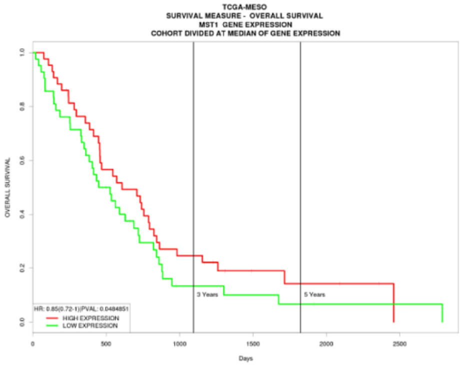

MST1

HAZARD RATIO	LCI (95%)	UCI (95%)	P VALUE
0.85	0.72	1	0.048485052524447

CATEGORY	SAMPLES	NO OF EVENTS	MEDIAN SURVIVAL	LOW CONF INT (95%)	UPP CONF INT (95%)
HIGH	43	35	608	457	844
LOW	42	38	488	361	727

C

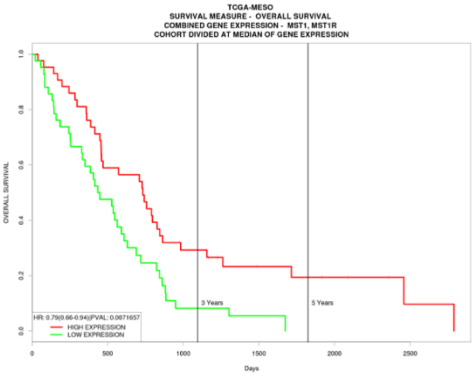

MST1 & MST1R

HAZARD RATIO	LCI (95%)	UCI (95%)	P VALUE
0.79	0.66	0.94	0.00716565135727054

CATEGORY	SAMPLES	NO OF EVENTS	MEDIAN SURVIVAL	LOW CONF INT (95%)	UPP CONF INT (95%)
HIGH	43	34	732	459	863
LOW	42	39	441	333	630

D

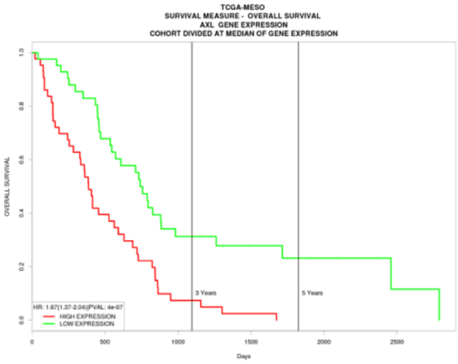

AXL

HAZARD RATIO	LCI (95%)	UCI (95%)	P VALUE
1.67	1.37	2.04	3.77137767237912e-07

CATEGORY	SAMPLES	NO OF EVENTS	MEDIAN SURVIVAL	LOW CONF INT (95%)	UPP CONF INT (95%)
HIGH	43	42	387	283	591
LOW	42	31	741	546	1261

Supplementary Figure 5

Figure S6. Analysis of macrophage infiltration and survival using macrophage marker, CD68.
(A) Immunohistochemical staining of an MPM TMA for macrophages using CD68 staining, at 10x and 20x magnification for a positive core.
(B) Following scoring by two pathologists and dichotomized closest to the median into low and high using >2, </=2 global scores, the results were analyzed using Cox regression for survival on (n=130) patients for which clinical data was available. No survival benefit was observed.
(C) Analysis of the mesothelioma TCGA dataset confirming that CD68 gene expression is not associated with any survival benefit in patients with MPM.

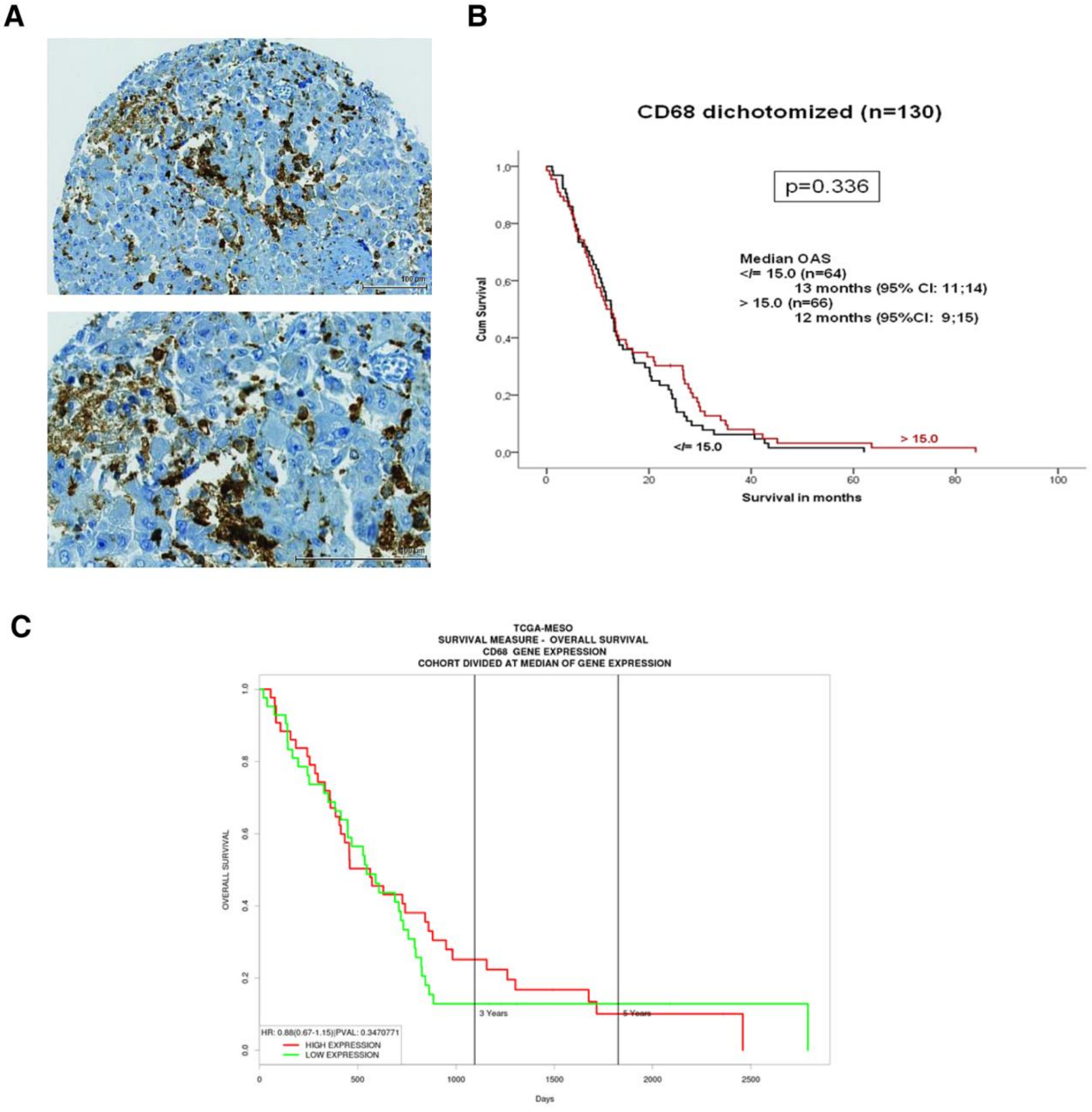

HAZARD RATIO	LCI (95%)	UCI (95%)	P VALUE
0.88	0.67	1.15	0.347077139856087

CATEGORY	SAMPLES	NO OF EVENTS	MEDIAN SURVIVAL	LOW CONF INT (95%)	UPP CONF INT (95%)
HIGH	43	37	563	406	881
LOW	42	36	546	448	757

Supplementary Figure 6

Figure S7: Recombinant MSP activates a number of downstream signaling pathways in the JU77 and NCI-H226 cell lines.

(A) JU77 cells were treated with recombinant human MST1 (250 ng/mL) for 0, 30 min, 24 h and 48 h (n=2). Protein was extracted and assayed on Phospho-Kinase Proteome Profiler arrays. Treatment values were compared with the 0 min time point to determine whether a protein was up or down regulated. An arbitrary cut-off of ± 20% change was set for increased or decreased activity, and the results are shown in tabulated form, with a representative image of the actual arrays shown. Yellow indicates increased phospho-kinase activity, while blue indicates decreased kinase activity. Grey indicates no significant change.

(B) An example of a Phospho-Kinase Proteome Profiler array result. Densitometric analysis was performed on arrays.

(C) A cross comparison of the effect of 30 min exposure of MST1 (250 ng/mL) on JU77 and NCI-H226, identified a series of kinases activated by MSP signaling across both cell lines.

Densitometric analysis was performed on arrays and the results are presented in tabulated form.

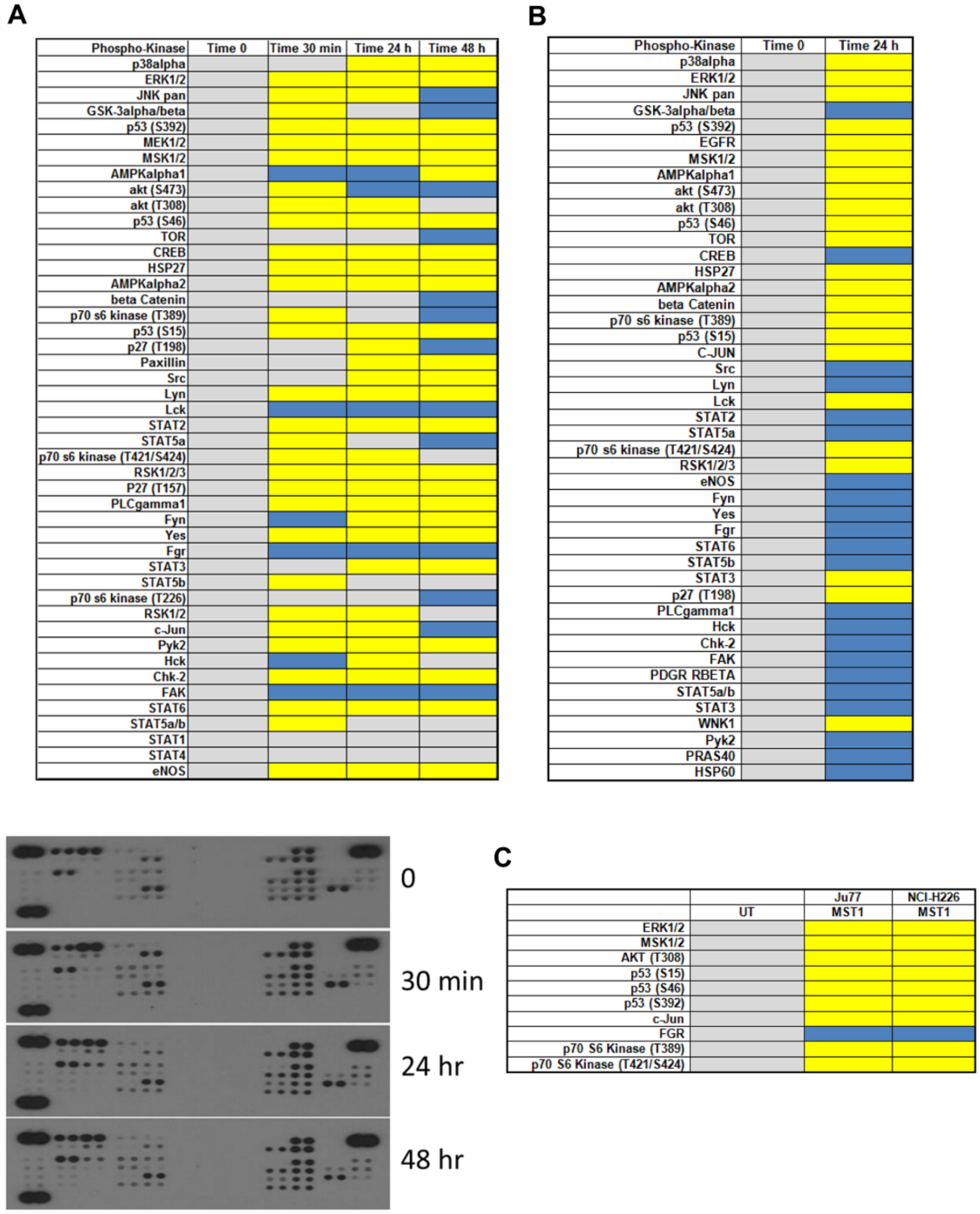

Supplementary Figure 7

Figure S8. Blockade of RON in MPM cell lines using various strategies. The effect of RON blockade on cellular proliferation was investigated on three cell lines (Met5A, JU77 and NCI-H226) using three different drugs (A) LCRF-0004 – a small molecule inhibitor, (B) NRWHE – a five amino acid peptide sequence and (C) (IMC)-RON8 – a humanized monoclonal antibody. The effect of recombinant human MST1 was also determined in the (D) Met5A and (E) JU77 cell lines. LCRF-0004 significantly decreased the proliferative capacity of the NCI-H226 cell lines, while MSP significantly decreased the proliferative capacity of the Met5A cells only. Significance was calculated based on a one-way ANOVA with a post-hoc Tukey's Multiple Comparison test (Significance relates to treatment vs. UT; *p<0.05; **p<0.01; ***p<0.001). UT – untreated.

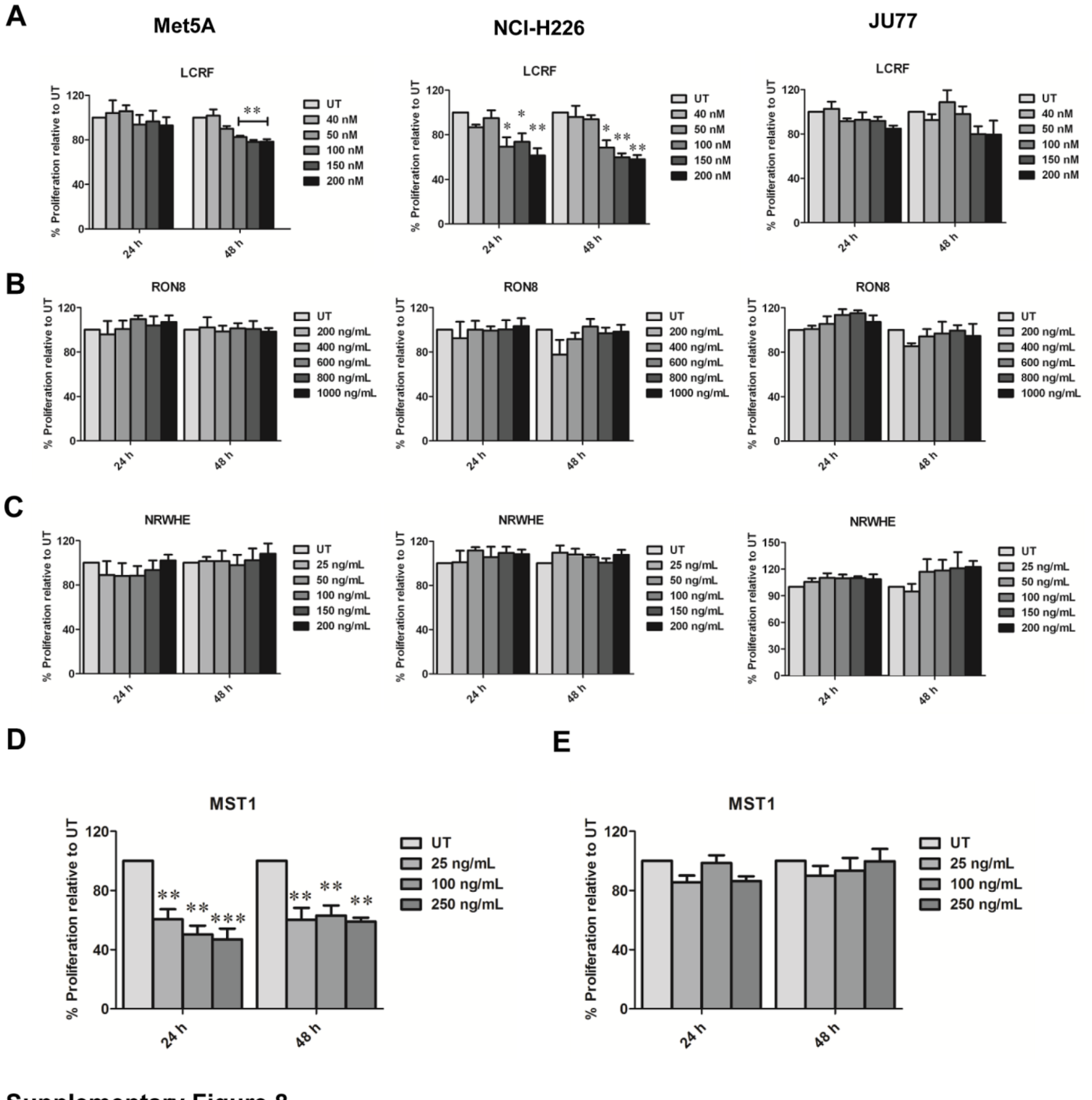

Supplementary Figure 8

Figure S9. Effects of LCRF-004 on cellular apoptosis or cell cycle progression as measured by HCS.

(A) LCRF-0004 (200 nM) significantly induces apoptosis in NCI-H226 cells as measured using a HCS based assay at 24 and 48 h (MST1 – 250 ng/mL). Significance was calculated based on a one-way ANOVA with a post-hoc Tukey's Multiple Comparison test. (* $p<0.05$; ** $p<0.01$; *** $p<0.001$).

(B) Drug treatment at both 24 and 48 h, promotes cells from <2n to the G0/G1 phase of the cell cycle. Data analyzed using a HCS based assay (LCRF – 200 nM; MST1 250 ng/mL).

A

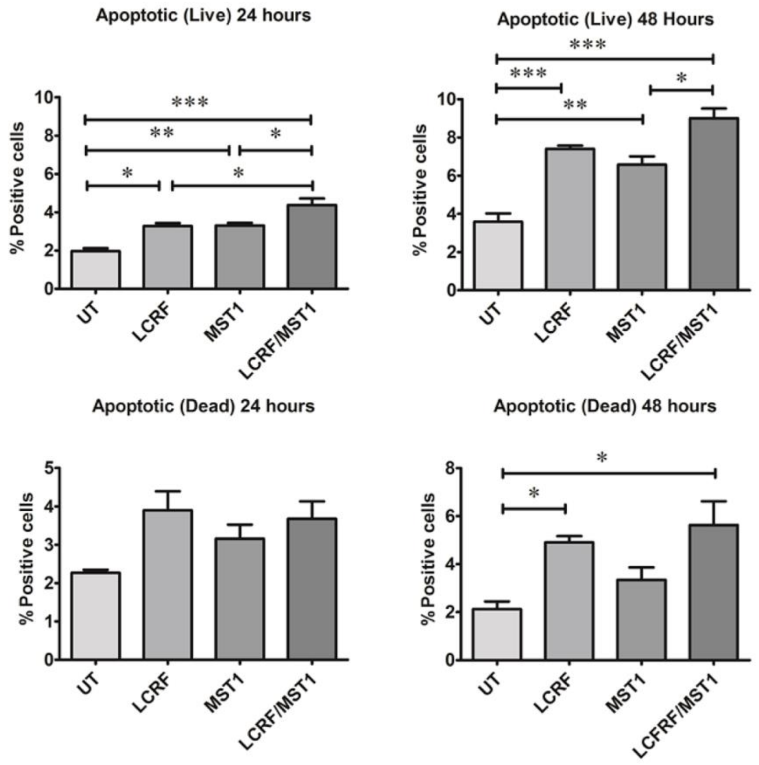

B

24 h	<2n	G0/G1	S	G2/M	>4n
UT	88.5	11.3	0.25	0.02	0.000
LCRF	63.4	35.7	0.77	0.07	0.022
MST1	87.4	12.2	0.29	0.03	0.007
Both	65.4	33.7	0.90	0.05	0.016

48 h	<2n	G0/G1	S	G2/M	>4n
UT	88.4	11.3	0.21	0.01	0.004
LCRF	67.2	30.4	2.30	0.09	0.041
MST1	86.6	12.9	0.38	0.02	0.018
Both	70.3	26.8	2.72	0.09	0.030

Figure S10. Expression of members of the MET/MST1R and TAM signaling pathway in NCI-H226 and Ju77.
mRNA levels of (A) flMST1R, sfMST1R and (B) MSP, GAS6, TAM (Tyro3, AXL, MERTK) Receptors and c-MET examined by standard end point PCR in NCI-H226 and Ju77 cells.

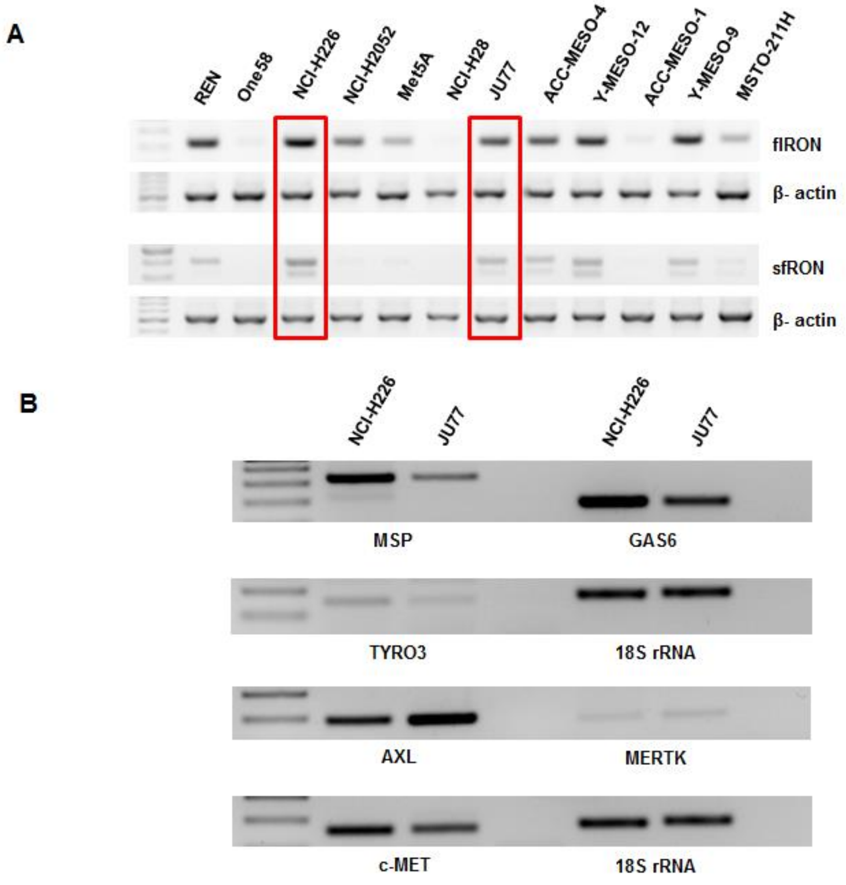

Supplementary Figure S10
